# Supplementary material for: Limited N-Glycan Processing Impacts Chaperone Expression Patterns, Cell Growth and Cell Invasiveness in Neuroblastoma
Source: Biology (Basel). 2023 Feb 11;12(2):293. doi: 10.3390/biology12020293 (PMC9953357; doi:10.3390/biology12020293)
Supplement: Supplementary file 1 [file biology-12-00293-s001.zip › biology-2186381-supplementary-S1.pdf]

No	Supp Table S1: NB_1<br>Structure	Glycan Mass	Relative Abundances (%)	Standard Deviation
1	$(Hex)_3$ $(HexNAc)_2$ $(Deoxyhexose)_1$	1345.6722	3.41	0.41
2	$(Hex)_4$ $(HexNAc)_2$	1375.6832	1.16	0.08
3	$(HexNAc)_1$ + $(Man)_3(GlcNAc)_2$	1416.7092	0.36	0.01
4	$(Hex)_2$ + $(Man)_3(GlcNAc)_2$	1579.7832	7.32	0.07
5	$(HexNAc)_1$ $(Deoxyhexose)_1$ + $(Man)_3(GlcNAc)_2$	1590.7992	1.05	0.01
6	$(Hex)_1$ $(HexNAc)_1$ + $(Man)_3(GlcNAc)_2$	1620.8092	0.65	0.55
7	$(HexNAc)_2$ + $(Man)_3(GlcNAc)_2$	1661.8362	0.38	0.05
8	$(Hex)_3$ + $(Man)_3(GlcNAc)_2$	1783.8822	20.09	2.11
9	$(Hex)_1$ $(HexNAc)_1$ $(Deoxyhexose)_1$ + $(Man)_3(GlcNAc)_2$	1794.8982	0.53	0.08
10	$(HexNAc)_2$ $(Deoxyhexose)_1$ + $(Man)_3(GlcNAc)_2$	1835.9252	2.43	0.37
11	$(HexNAc)_3$ + $(Man)_3(GlcNAc)_2$	1906.9622	0.17	0.02
12	$(HexNAc)_1$ $(Deoxyhexose)_1$ $(NeuAc)_1$ + $(Man)_2(GlcNAc)_2$	1951.9722	0.03	0.01
13	$(Hex)_1$ $(HexNAc)_1$ $(NeuAc)_1$ + $(Man)_3(GlcNAc)_2$	1981.9832	0.48	0.11
14	$(Hex)_4$ + $(Man)_3(GlcNAc)_2$	1987.9822	12.11	0.23
15	$(Hex)_1$ $(HexNAc)_2$ $(Deoxyhexose)_1$ + $(Man)_3(GlcNAc)_2$	2040.0252	0.53	0.04
16	$(HexNAc)_3$ $(Deoxyhexose)_1$ + $(Man)_3(GlcNAc)_2$	2081.0512	0.72	0.09
17	$(Hex)_1$ $(HexNAc)_1$ $(Deoxyhexose)_1$ $(NeuAc)_1$ + $(Man)_3(GlcNAc)_2$	2156.0722	1.65	0.09
18	$(Hex)_2$ $(HexNAc)_1$ $(NeuAc)_1$ + $(Man)_3(GlcNAc)_2$	2186.0832	1.55	0.40
19	$(Hex)_5$ + $(Man)_3(GlcNAc)_2$	2192.0822	14.78	2.84
20	$(Hex)_1$ $(HexNAc)_2$ $(NeuAc)_1$ + $(Man)_3(GlcNAc)_2$	2227.1092	0.28	0.06
21	$(Hex)_2$ $(HexNAc)_2$ $(Deoxyhexose)_1$ + $(Man)_3(GlcNAc)_2$	2244.1242	0.29	0.08
22	$(Hex)_1$ $(HexNAc)_3$ $(Deoxyhexose)_1$ + $(Man)_3(GlcNAc)_2$	2285.1512	0.28	0.13
23	$(Hex)_1$ $(HexNAc)_1$ $(Deoxyhexose)_2$ $(NeuAc)_1$ + $(Man)_3(GlcNAc)_2$	2330.1612	0.14	0.02
24	$(Hex)_2$ $(HexNAc)_1$ $(Deoxyhexose)_1$ $(NeuAc)_1$ + $(Man)_3(GlcNAc)_2$	2360.1722	2.83	0.33
25	$(Hex)_3$ $(HexNAc)_1$ $(NeuAc)_1$ + $(Man)_3(GlcNAc)_2$	2390.1822	2.29	0.12
26	$(Hex)_6$ + $(Man)_3(GlcNAc)_2$	2396.1822	9.73	1.24
27	$(Hex)_1$ $(HexNAc)_2$ $(Deoxyhexose)_1$ $(NeuAc)_1$ + $(Man)_3(GlcNAc)_2$	2401.1982	1.51	0.21
28	$(Hex)_2$ $(HexNAc)_2$ $(NeuAc)_1$ + $(Man)_3(GlcNAc)_2$	2431.2092	0.46	0.05
29	$(Hex)_2$ $(HexNAc)_3$ $(Deoxyhexose)_1$ + $(Man)_3(GlcNAc)_2$	2489.2512	0.07	0.01
30	$(Hex)_3$ $(HexNAc)_1$ $(Deoxyhexose)_1$ $(NeuAc)_1$ + $(Man)_3(GlcNAc)_2$	2564.2722	0.91	0.02
31	$(Hex)_2$ $(HexNAc)_2$ $(Deoxyhexose)_1$ $(NeuAc)_1$ + $(Man)_3(GlcNAc)_2$	2605.2982	1.35	0.09
32	$(Hex)_3$ $(HexNAc)_2$ $(NeuAc)_1$ + $(Man)_3(GlcNAc)_2$	2635.3092	0.26	0.01
33	$(Hex)_1$ $(HexNAc)_3$ $(Deoxyhexose)_1$ $(NeuAc)_1$ + $(Man)_3(GlcNAc)_2$	2646.3252	0.37	0.07
34	$(Hex)_2$ $(HexNAc)_2$ $(Deoxyhexose)_2$ $(NeuAc)_1$ + $(Man)_3(GlcNAc)_2$	2779.3872	0.72	0.09
35	$(Hex)_2$ $(HexNAc)_2$ $(NeuAc)_2$ + $(Man)_3(GlcNAc)_2$	2792.3832	0.95	0.04
36	$(Hex)_3$ $(HexNAc)_2$ $(Deoxyhexose)_1$ $(NeuAc)_1$ + $(Man)_3(GlcNAc)_2$	2809.3982	0.80	0.01
37	$(Hex)_2$ $(HexNAc)_3$ $(Deoxyhexose)_1$ $(NeuAc)_1$ + $(Man)_3(GlcNAc)_2$	2850.4242	0.19	0.01
38	$(Hex)_2$ $(HexNAc)_2$ $(Deoxyhexose)_1$ $(NeuAc)_2$ + $(Man)_3(GlcNAc)_2$	2966.4722	4.20	0.25
39	$(Hex)_3$ $(HexNAc)_2$ $(Deoxyhexose)_2$ $(NeuAc)_1$ + $(Man)_3(GlcNAc)_2$	2983.4872	0.15	0.00
40	$(Hex)_2$ $(HexNAc)_3$ $(NeuAc)_2$ + $(Man)_3(GlcNAc)_2$	3037.5092	0.01	0.01
41	$(Hex)_2$ $(HexNAc)_2$ $(Deoxyhexose)_2$ $(NeuAc)_2$ + $(Man)_3(GlcNAc)_2$	3140.5612	0.24	0.01
42	$(Hex)_2$ $(HexNAc)_3$ $(Deoxyhexose)_1$ $(NeuAc)_2$ + $(Man)_3(GlcNAc)_2$	3211.5982	0.46	0.03
43	$(Hex)_3$ $(HexNAc)_3$ $(NeuAc)_2$ + $(Man)_3(GlcNAc)_2$	3241.6092	0.09	0.01
44	$(Hex)_3$ $(HexNAc)_3$ $(Deoxyhexose)_1$ $(NeuAc)_2$ + $(Man)_3(GlcNAc)_2$	3415.6982	0.31	0.00
45	$(Hex)_3$ $(HexNAc)_3$ $(NeuAc)_3$ + $(Man)_3(GlcNAc)_2$	3602.7822	0.29	0.01
46	$(Hex)_4$ $(HexNAc)_3$ $(Deoxyhexose)_1$ $(NeuAc)_2$ + $(Man)_3(GlcNAc)_2$	3619.7982	0.15	0.01
47	$(Hex)_3$ $(HexNAc)_3$ $(Deoxyhexose)_1$ $(NeuAc)_3$ + $(Man)_3(GlcNAc)_2$	3776.8722	1.27	0.05

	Supp Table S2: NB_1(-*Mgat1*)		Relative Abundances (%)	Standard Deviation
No.	Structure	Glycan Mass		
1	$(Hex)_3$ $(HexNAc)_2$ $(Deoxyhexose)_1$	1345.6722	0.06	0.00
2	$(Hex)_4$ $(HexNAc)_2$	1375.6832	4.07	0.17
3	$(HexNAc)_1$ + $(Man)_3(GlcNAc)_2$	1416.7092	0.01	0.00
4	$(Hex)_2$ + $(Man)_3(GlcNAc)_2$	1579.7832	62.52	0.62
5	$(HexNAc)_1$ $(Deoxyhexose)_1$ + $(Man)_3(GlcNAc)_2$	1590.7992	0.03	0.00
6	$(Hex)_1$ $(HexNAc)_1$ + $(Man)_3(GlcNAc)_2$	1620.8092	0.02	0.00
7	$(HexNAc)_2$ + $(Man)_3(GlcNAc)_2$	1661.8362	0.00	0.00
8	$(Hex)_3$ + $(Man)_3(GlcNAc)_2$	1783.8822	10.97	0.72
9	$(Hex)_1$ $(HexNAc)_1$ $(Deoxyhexose)_1$ + $(Man)_3(GlcNAc)_2$	1794.8982	0.01	0.00
10	$(HexNAc)_2$ $(Deoxyhexose)_1$ + $(Man)_3(GlcNAc)_2$	1835.9252	0.01	0.00
11	$(HexNAc)_3$ + $(Man)_3(GlcNAc)_2$	1906.9622	0.01	0.00
12	$(HexNAc)_1$ $(Deoxyhexose)_1$ $(NeuAc)_1$ + $(Man)_2(GlcNAc)_2$	1951.9722	0.00	0.00
13	$(Hex)_1$ $(HexNAc)_1$ $(NeuAc)_1$ + $(Man)_3(GlcNAc)_2$	1981.9832	0.03	0.01
14	$(Hex)_4$ + $(Man)_3(GlcNAc)_2$	1987.9822	7.86	0.25
15	$(Hex)_1$ $(HexNAc)_2$ $(Deoxyhexose)_1$ + $(Man)_3(GlcNAc)_2$	2040.0252	0.00	0.00
16	$(HexNAc)_3$ $(Deoxyhexose)_1$ + $(Man)_3(GlcNAc)_2$	2081.0512	0.00	0.00
17	$(Hex)_1$ $(HexNAc)_1$ $(Deoxyhexose)_1$ $(NeuAc)_1$ + $(Man)_3(GlcNAc)_2$	2156.0722	0.87	0.07
18	$(Hex)_2$ $(HexNAc)_1$ $(NeuAc)_1$ + $(Man)_3(GlcNAc)_2$	2186.0832	0.06	0.00
19	$(Hex)_5$ + $(Man)_3(GlcNAc)_2$	2192.0822	8.62	0.08
20	$(Hex)_1$ $(HexNAc)_2$ $(NeuAc)_1$ + $(Man)_3(GlcNAc)_2$	2227.1092	0.01	0.00
21	$(Hex)_2$ $(HexNAc)_2$ $(Deoxyhexose)_1$ + $(Man)_3(GlcNAc)_2$	2244.1242	0.01	0.00
22	$(Hex)_1$ $(HexNAc)_3$ $(Deoxyhexose)_1$ + $(Man)_3(GlcNAc)_2$	2285.1512	0.00	0.00
23	$(Hex)_1$ $(HexNAc)_1$ $(Deoxyhexose)_2$ $(NeuAc)_1$ + $(Man)_3(GlcNAc)_2$	2330.1612	0.00	0.00
24	$(Hex)_2$ $(HexNAc)_1$ $(Deoxyhexose)_1$ $(NeuAc)_1$ + $(Man)_3(GlcNAc)_2$	2360.1722	0.00	0.00
25	$(Hex)_3$ $(HexNAc)_1$ $(NeuAc)_1$ + $(Man)_3(GlcNAc)_2$	2390.1822	0.13	0.00
26	$(Hex)_6$ + $(Man)_3(GlcNAc)_2$	2396.1822	4.27	0.24
27	$(Hex)_1$ $(HexNAc)_2$ $(Deoxyhexose)_1$ $(NeuAc)_1$ + $(Man)_3(GlcNAc)_2$	2401.1982	0.00	0.00
28	$(Hex)_2$ $(HexNAc)_2$ $(NeuAc)_1$ + $(Man)_3(GlcNAc)_2$	2431.2092	0.04	0.00
29	$(Hex)_2$ $(HexNAc)_3$ $(Deoxyhexose)_1$ + $(Man)_3(GlcNAc)_2$	2489.2512	0.00	0.00
30	$(Hex)_3$ $(HexNAc)_1$ $(Deoxyhexose)_1$ $(NeuAc)_1$ + $(Man)_3(GlcNAc)_2$	2564.2722	0.05	0.00
31	$(Hex)_2$ $(HexNAc)_2$ $(Deoxyhexose)_1$ $(NeuAc)_1$ + $(Man)_3(GlcNAc)_2$	2605.2982	0.01	0.00
32	$(Hex)_3$ $(HexNAc)_2$ $(NeuAc)_1$ + $(Man)_3(GlcNAc)_2$	2635.3092	0.00	0.00
33	$(Hex)_1$ $(HexNAc)_3$ $(Deoxyhexose)_1$ $(NeuAc)_1$ + $(Man)_3(GlcNAc)_2$	2646.3252	0.11	0.02
34	$(Hex)_2$ $(HexNAc)_2$ $(Deoxyhexose)_2$ $(NeuAc)_1$ + $(Man)_3(GlcNAc)_2$	2779.3872	0.00	0.00
35	$(Hex)_2$ $(HexNAc)_2$ $(NeuAc)_2$ + $(Man)_3(GlcNAc)_2$	2792.3832	0.08	0.00
36	$(Hex)_3$ $(HexNAc)_2$ $(Deoxyhexose)_1$ $(NeuAc)_1$ + $(Man)_3(GlcNAc)_2$	2809.3982	0.01	0.00
37	$(Hex)_2$ $(HexNAc)_3$ $(Deoxyhexose)_1$ $(NeuAc)_1$ + $(Man)_3(GlcNAc)_2$	2850.4242	0.02	0.00
38	$(Hex)_2$ $(HexNAc)_2$ $(Deoxyhexose)_1$ $(NeuAc)_2$ + $(Man)_3(GlcNAc)_2$	2966.4722	0.03	0.01
39	$(Hex)_3$ $(HexNAc)_2$ $(Deoxyhexose)_2$ $(NeuAc)_1$ + $(Man)_3(GlcNAc)_2$	2983.4872	0.00	0.00
40	$(Hex)_2$ $(HexNAc)_3$ $(NeuAc)_2$ + $(Man)_3(GlcNAc)_2$	3037.5092	0.00	0.00
41	$(Hex)_2$ $(HexNAc)_2$ $(Deoxyhexose)_2$ $(NeuAc)_2$ + $(Man)_3(GlcNAc)_2$	3140.5612	0.00	0.00
42	$(Hex)_2$ $(HexNAc)_3$ $(Deoxyhexose)_1$ $(NeuAc)_2$ + $(Man)_3(GlcNAc)_2$	3211.5982	0.01	0.00
43	$(Hex)_3$ $(HexNAc)_3$ $(NeuAc)_2$ + $(Man)_3(GlcNAc)_2$	3241.6092	0.01	0.00
44	$(Hex)_3$ $(HexNAc)_3$ $(Deoxyhexose)_1$ $(NeuAc)_2$ + $(Man)_3(GlcNAc)_2$	3415.6982	0.00	0.00
45	$(Hex)_3$ $(HexNAc)_3$ $(NeuAc)_3$ + $(Man)_3(GlcNAc)_2$	3602.7822	0.02	0.00
46	$(Hex)_4$ $(HexNAc)_3$ $(Deoxyhexose)_1$ $(NeuAc)_2$ + $(Man)_3(GlcNAc)_2$	3619.7982	0.00	0.00
47	$(Hex)_3$ $(HexNAc)_3$ $(Deoxyhexose)_1$ $(NeuAc)_3$ + $(Man)_3(GlcNAc)_2$	3776.8722	0.00	0.00

No.	Supp Table S3: NB_1(-*Mgat2)*<br>Structure	Glycan Mass	Relative Abundances (%)	Standard Deviation
1	$(Hex)_3$ $(HexNAc)_2$ $(Deoxyhexose)_1$	1345.6722	5.99	0.08
2	$(Hex)_4$ $(HexNAc)_2$	1375.6832	1.33	0.05
3	$(HexNAc)_1$ + $(Man)_3(GlcNAc)_2$	1416.7092	0.31	0.00
4	$(Hex)_2$ + $(Man)_3(GlcNAc)_2$	1579.7832	7.91	0.57
5	$(HexNAc)_1$ $(Deoxyhexose)_1$ + $(Man)_3(GlcNAc)_2$	1590.7992	2.41	0.21
6	$(Hex)_1$ $(HexNAc)_1$ + $(Man)_3(GlcNAc)_2$	1620.8092	0.17	0.03
7	$(HexNAc)_2$ + $(Man)_3(GlcNAc)_2$	1661.8362	0.12	0.01
8	$(Hex)_3$ + $(Man)_3(GlcNAc)_2$	1783.8822	14.22	0.35
9	$(Hex)_1$ $(HexNAc)_1$ $(Deoxyhexose)_1$ + $(Man)_3(GlcNAc)_2$	1794.8982	0.52	0.01
10	$(HexNAc)_2$ $(Deoxyhexose)_1$ + $(Man)_3(GlcNAc)_2$	1835.9252	0.70	0.09
11	$(HexNAc)_3$ + $(Man)_3(GlcNAc)_2$	1906.9622	0.06	0.03
12	$(HexNAc)_1$ $(Deoxyhexose)_1$ $(NeuAc)_1$ + $(Man)_2(GlcNAc)_2$	1951.9722	0.16	0.02
13	$(Hex)_1$ $(HexNAc)_1$ $(NeuAc)_1$ + $(Man)_3(GlcNAc)_2$	1981.9832	2.81	0.14
14	$(Hex)_4$ + $(Man)_3(GlcNAc)_2$	1987.9822	10.39	0.54
15	$(Hex)_1$ $(HexNAc)_2$ $(Deoxyhexose)_1$ + $(Man)_3(GlcNAc)_2$	2040.0252	2.59	0.04
16	$(HexNAc)_3$ $(Deoxyhexose)_1$ + $(Man)_3(GlcNAc)_2$	2081.0512	0.01	0.01
17	$(Hex)_1$ $(HexNAc)_1$ $(Deoxyhexose)_1$ $(NeuAc)_1$ + $(Man)_3(GlcNAc)_2$	2156.0722	19.90	0.45
18	$(Hex)_2$ $(HexNAc)_1$ $(NeuAc)_1$ + $(Man)_3(GlcNAc)_2$	2186.0832	1.51	0.05
19	$(Hex)_5$ + $(Man)_3(GlcNAc)_2$	2192.0822	12.05	0.70
20	$(Hex)_1$ $(HexNAc)_2$ $(NeuAc)_1$ + $(Man)_3(GlcNAc)_2$	2227.1092	0.09	0.00
21	$(Hex)_2$ $(HexNAc)_2$ $(Deoxyhexose)_1$ + $(Man)_3(GlcNAc)_2$	2244.1242	0.67	0.04
22	$(Hex)_1$ $(HexNAc)_3$ $(Deoxyhexose)_1$ + $(Man)_3(GlcNAc)_2$	2285.1512	0.01	0.00
23	$(Hex)_1$ $(HexNAc)_1$ $(Deoxyhexose)_2$ $(NeuAc)_1$ + $(Man)_3(GlcNAc)_2$	2330.1612	0.69	0.03
24	$(Hex)_2$ $(HexNAc)_1$ $(Deoxyhexose)_1$ $(NeuAc)_1$ + $(Man)_3(GlcNAc)_2$	2360.1722	1.06	0.09
25	$(Hex)_3$ $(HexNAc)_1$ $(NeuAc)_1$ + $(Man)_3(GlcNAc)_2$	2390.1822	1.26	0.06
26	$(Hex)_6$ + $(Man)_3(GlcNAc)_2$	2396.1822	6.99	0.00
27	$(Hex)_1$ $(HexNAc)_2$ $(Deoxyhexose)_1$ $(NeuAc)_1$ + $(Man)_3(GlcNAc)_2$	2401.1982	0.59	0.02
28	$(Hex)_2$ $(HexNAc)_2$ $(NeuAc)_1$ + $(Man)_3(GlcNAc)_2$	2431.2092	0.07	0.00
29	$(Hex)_2$ $(HexNAc)_3$ $(Deoxyhexose)_1$ + $(Man)_3(GlcNAc)_2$	2489.2512	0.00	0.00
30	$(Hex)_3$ $(HexNAc)_1$ $(Deoxyhexose)_1$ $(NeuAc)_1$ + $(Man)_3(GlcNAc)_2$	2564.2722	0.46	0.03
31	$(Hex)_2$ $(HexNAc)_2$ $(Deoxyhexose)_1$ $(NeuAc)_1$ + $(Man)_3(GlcNAc)_2$	2605.2982	0.58	0.03
32	$(Hex)_3$ $(HexNAc)_2$ $(NeuAc)_1$ + $(Man)_3(GlcNAc)_2$	2635.3092	0.01	0.00
33	$(Hex)_1$ $(HexNAc)_3$ $(Deoxyhexose)_1$ $(NeuAc)_1$ + $(Man)_3(GlcNAc)_2$	2646.3252	0.14	0.01
34	$(Hex)_2$ $(HexNAc)_2$ $(Deoxyhexose)_2$ $(NeuAc)_1$ + $(Man)_3(GlcNAc)_2$	2779.3872	0.10	0.00
35	$(Hex)_2$ $(HexNAc)_2$ $(NeuAc)_2$ + $(Man)_3(GlcNAc)_2$	2792.3832	0.31	0.04
36	$(Hex)_3$ $(HexNAc)_2$ $(Deoxyhexose)_1$ $(NeuAc)_1$ + $(Man)_3(GlcNAc)_2$	2809.3982	0.07	0.00
37	$(Hex)_2$ $(HexNAc)_3$ $(Deoxyhexose)_1$ $(NeuAc)_1$ + $(Man)_3(GlcNAc)_2$	2850.4242	0.10	0.01
38	$(Hex)_2$ $(HexNAc)_2$ $(Deoxyhexose)_1$ $(NeuAc)_2$ + $(Man)_3(GlcNAc)_2$	2966.4722	3.10	0.20
39	$(Hex)_3$ $(HexNAc)_2$ $(Deoxyhexose)_2$ $(NeuAc)_1$ + $(Man)_3(GlcNAc)_2$	2983.4872	0.03	0.01
40	$(Hex)_2$ $(HexNAc)_3$ $(NeuAc)_2$ + $(Man)_3(GlcNAc)_2$	3037.5092	0.00	0.00
41	$(Hex)_2$ $(HexNAc)_2$ $(Deoxyhexose)_2$ $(NeuAc)_2$ + $(Man)_3(GlcNAc)_2$	3140.5612	0.08	0.01
42	$(Hex)_2$ $(HexNAc)_3$ $(Deoxyhexose)_1$ $(NeuAc)_2$ + $(Man)_3(GlcNAc)_2$	3211.5982	0.09	0.00
43	$(Hex)_3$ $(HexNAc)_3$ $(NeuAc)_2$ + $(Man)_3(GlcNAc)_2$	3241.6092	0.02	0.00
44	$(Hex)_3$ $(HexNAc)_3$ $(Deoxyhexose)_1$ $(NeuAc)_2$ + $(Man)_3(GlcNAc)_2$	3415.6982	0.16	0.01
45	$(Hex)_3$ $(HexNAc)_3$ $(NeuAc)_3$ + $(Man)_3(GlcNAc)_2$	3602.7822	0.07	0.00
46	$(Hex)_4$ $(HexNAc)_3$ $(Deoxyhexose)_1$ $(NeuAc)_2$ + $(Man)_3(GlcNAc)_2$	3619.7982	0.00	0.00
47	$(Hex)_3$ $(HexNAc)_3$ $(Deoxyhexose)_1$ $(NeuAc)_3$ + $(Man)_3(GlcNAc)_2$	3776.8722	0.08	0.01

No.	Supp Table S4: NB_1(-*Mgat3*)<br>Structure	Glycan Mass	Relative Abundances (%)	Standard Deviation
1	$(Hex)_3$ $(HexNAc)_2$ $(Deoxyhexose)_1$	1345.6722	2.96	0.16
2	$(Hex)_4$ $(HexNAc)_2$	1375.6832	1.13	0.14
3	$(HexNAc)_1$ + $(Man)_3(GlcNAc)_2$	1416.7092	0.25	0.02
4	$(Hex)_2$ + $(Man)_3(GlcNAc)_2$	1579.7832	7.45	1.09
5	$(HexNAc)_1$ $(Deoxyhexose)_1$ + $(Man)_3(GlcNAc)_2$	1590.7992	0.83	0.16
6	$(Hex)_1$ $(HexNAc)_1$ + $(Man)_3(GlcNAc)_2$	1620.8092	0.23	0.05
7	$(HexNAc)_2$ + $(Man)_3(GlcNAc)_2$	1661.8362	0.27	0.05
8	$(Hex)_3$ + $(Man)_3(GlcNAc)_2$	1783.8822	23.94	1.04
9	$(Hex)_1$ $(HexNAc)_1$ $(Deoxyhexose)_1$ + $(Man)_3(GlcNAc)_2$	1794.8982	0.45	0.06
10	$(HexNAc)_2$ $(Deoxyhexose)_1$ + $(Man)_3(GlcNAc)_2$	1835.9252	1.63	0.31
11	$(HexNAc)_3$ + $(Man)_3(GlcNAc)_2$	1906.9622	0.08	0.02
12	$(HexNAc)_1$ $(Deoxyhexose)_1$ $(NeuAc)_1$ + $(Man)_2(GlcNAc)_2$	1951.9722	0.04	0.02
13	$(Hex)_1$ $(HexNAc)_1$ $(NeuAc)_1$ + $(Man)_3(GlcNAc)_2$	1981.9832	0.30	0.04
14	$(Hex)_4$ + $(Man)_3(GlcNAc)_2$	1987.9822	15.17	0.69
15	$(Hex)_1$ $(HexNAc)_2$ $(Deoxyhexose)_1$ + $(Man)_3(GlcNAc)_2$	2040.0252	0.45	0.06
16	$(HexNAc)_3$ $(Deoxyhexose)_1$ + $(Man)_3(GlcNAc)_2$	2081.0512	0.41	0.10
17	$(Hex)_1$ $(HexNAc)_1$ $(Deoxyhexose)_1$ $(NeuAc)_1$ + $(Man)_3(GlcNAc)_2$	2156.0722	1.04	0.05
18	$(Hex)_2$ $(HexNAc)_1$ $(NeuAc)_1$ + $(Man)_3(GlcNAc)_2$	2186.0832	1.07	0.01
19	$(Hex)_5$ + $(Man)_3(GlcNAc)_2$	2192.0822	15.61	0.12
20	$(Hex)_1$ $(HexNAc)_2$ $(NeuAc)_1$ + $(Man)_3(GlcNAc)_2$	2227.1092	0.20	0.01
21	$(Hex)_2$ $(HexNAc)_2$ $(Deoxyhexose)_1$ + $(Man)_3(GlcNAc)_2$	2244.1242	0.23	0.00
22	$(Hex)_1$ $(HexNAc)_3$ $(Deoxyhexose)_1$ + $(Man)_3(GlcNAc)_2$	2285.1512	0.04	0.01
23	$(Hex)_1$ $(HexNAc)_1$ $(Deoxyhexose)_2$ $(NeuAc)_1$ + $(Man)_3(GlcNAc)_2$	2330.1612	0.03	0.01
24	$(Hex)_2$ $(HexNAc)_1$ $(Deoxyhexose)_1$ $(NeuAc)_1$ + $(Man)_3(GlcNAc)_2$	2360.1722	1.85	0.22
25	$(Hex)_3$ $(HexNAc)_1$ $(NeuAc)_1$ + $(Man)_3(GlcNAc)_2$	2390.1822	2.84	0.44
26	$(Hex)_6$ + $(Man)_3(GlcNAc)_2$	2396.1822	8.25	0.53
27	$(Hex)_1$ $(HexNAc)_2$ $(Deoxyhexose)_1$ $(NeuAc)_1$ + $(Man)_3(GlcNAc)_2$	2401.1982	1.18	0.06
28	$(Hex)_2$ $(HexNAc)_2$ $(NeuAc)_1$ + $(Man)_3(GlcNAc)_2$	2431.2092	0.38	0.01
29	$(Hex)_2$ $(HexNAc)_3$ $(Deoxyhexose)_1$ + $(Man)_3(GlcNAc)_2$	2489.2512	0.02	0.00
30	$(Hex)_3$ $(HexNAc)_1$ $(Deoxyhexose)_1$ $(NeuAc)_1$ + $(Man)_3(GlcNAc)_2$	2564.2722	0.96	0.01
31	$(Hex)_2$ $(HexNAc)_2$ $(Deoxyhexose)_1$ $(NeuAc)_1$ + $(Man)_3(GlcNAc)_2$	2605.2982	1.34	0.04
32	$(Hex)_3$ $(HexNAc)_2$ $(NeuAc)_1$ + $(Man)_3(GlcNAc)_2$	2635.3092	0.24	0.01
33	$(Hex)_1$ $(HexNAc)_3$ $(Deoxyhexose)_1$ $(NeuAc)_1$ + $(Man)_3(GlcNAc)_2$	2646.3252	0.21	0.01
34	$(Hex)_2$ $(HexNAc)_2$ $(Deoxyhexose)_2$ $(NeuAc)_1$ + $(Man)_3(GlcNAc)_2$	2779.3872	0.88	0.12
35	$(Hex)_2$ $(HexNAc)_2$ $(NeuAc)_2$ + $(Man)_3(GlcNAc)_2$	2792.3832	1.13	0.07
36	$(Hex)_3$ $(HexNAc)_2$ $(Deoxyhexose)_1$ $(NeuAc)_1$ + $(Man)_3(GlcNAc)_2$	2809.3982	0.98	0.07
37	$(Hex)_2$ $(HexNAc)_3$ $(Deoxyhexose)_1$ $(NeuAc)_1$ + $(Man)_3(GlcNAc)_2$	2850.4242	0.07	0.01
38	$(Hex)_2$ $(HexNAc)_2$ $(Deoxyhexose)_1$ $(NeuAc)_2$ + $(Man)_3(GlcNAc)_2$	2966.4722	4.10	0.36
39	$(Hex)_3$ $(HexNAc)_2$ $(Deoxyhexose)_2$ $(NeuAc)_1$ + $(Man)_3(GlcNAc)_2$	2983.4872	0.12	0.02
40	$(Hex)_2$ $(HexNAc)_3$ $(NeuAc)_2$ + $(Man)_3(GlcNAc)_2$	3037.5092	0.02	0.00
41	$(Hex)_2$ $(HexNAc)_2$ $(Deoxyhexose)_2$ $(NeuAc)_2$ + $(Man)_3(GlcNAc)_2$	3140.5612	0.23	0.02
42	$(Hex)_2$ $(HexNAc)_3$ $(Deoxyhexose)_1$ $(NeuAc)_2$ + $(Man)_3(GlcNAc)_2$	3211.5982	0.14	0.01
43	$(Hex)_3$ $(HexNAc)_3$ $(NeuAc)_2$ + $(Man)_3(GlcNAc)_2$	3241.6092	0.07	0.00
44	$(Hex)_3$ $(HexNAc)_3$ $(Deoxyhexose)_1$ $(NeuAc)_2$ + $(Man)_3(GlcNAc)_2$	3415.6982	0.25	0.03
45	$(Hex)_3$ $(HexNAc)_3$ $(NeuAc)_3$ + $(Man)_3(GlcNAc)_2$	3602.7822	0.22	0.01
46	$(Hex)_4$ $(HexNAc)_3$ $(Deoxyhexose)_1$ $(NeuAc)_2$ + $(Man)_3(GlcNAc)_2$	3619.7982	0.10	0.00
47	$(Hex)_3$ $(HexNAc)_3$ $(Deoxyhexose)_1$ $(NeuAc)_3$ + $(Man)_3(GlcNAc)_2$	3776.8722	0.62	0.04

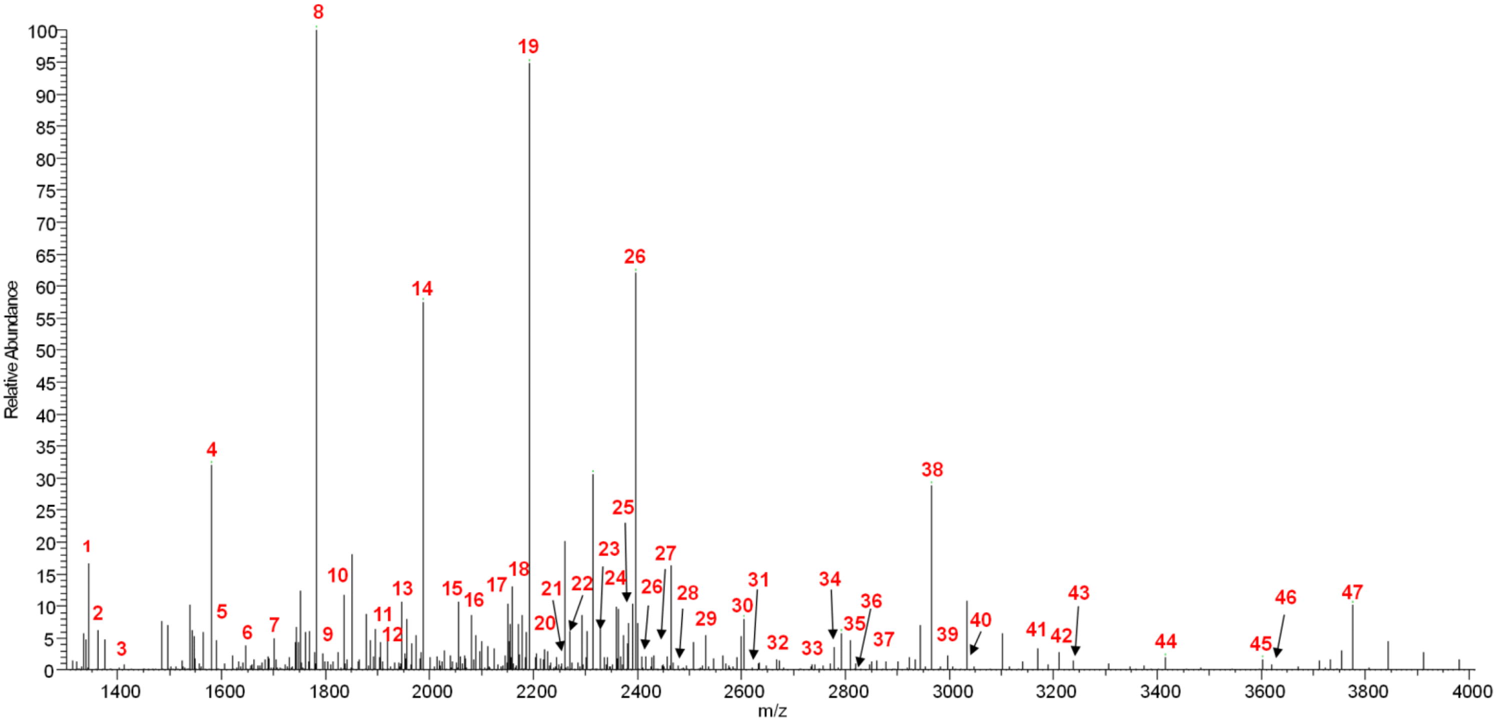

Supp Figure S1: Averaged and deconvoluted LC-ESI-MS spectrum of N-glycans released from NB_1. N-glycans are labelled with numbers corresponding to Supp Table S1.

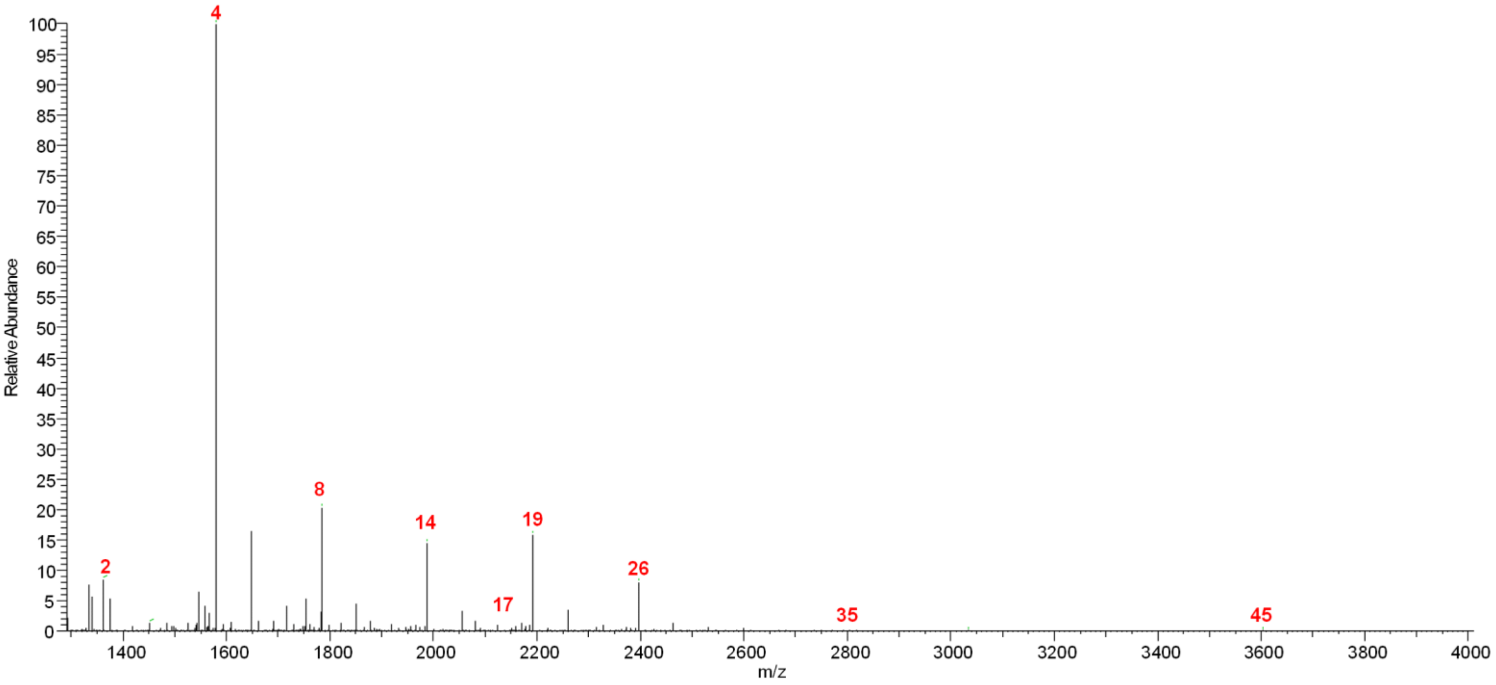

Supp Figure S2: Averaged and deconvoluted LC-ESI-MS spectrum of N-glycans released from NB_1(-*Mgat1*). N-glycans are labelled with numbers corresponding to Supp Table S2.

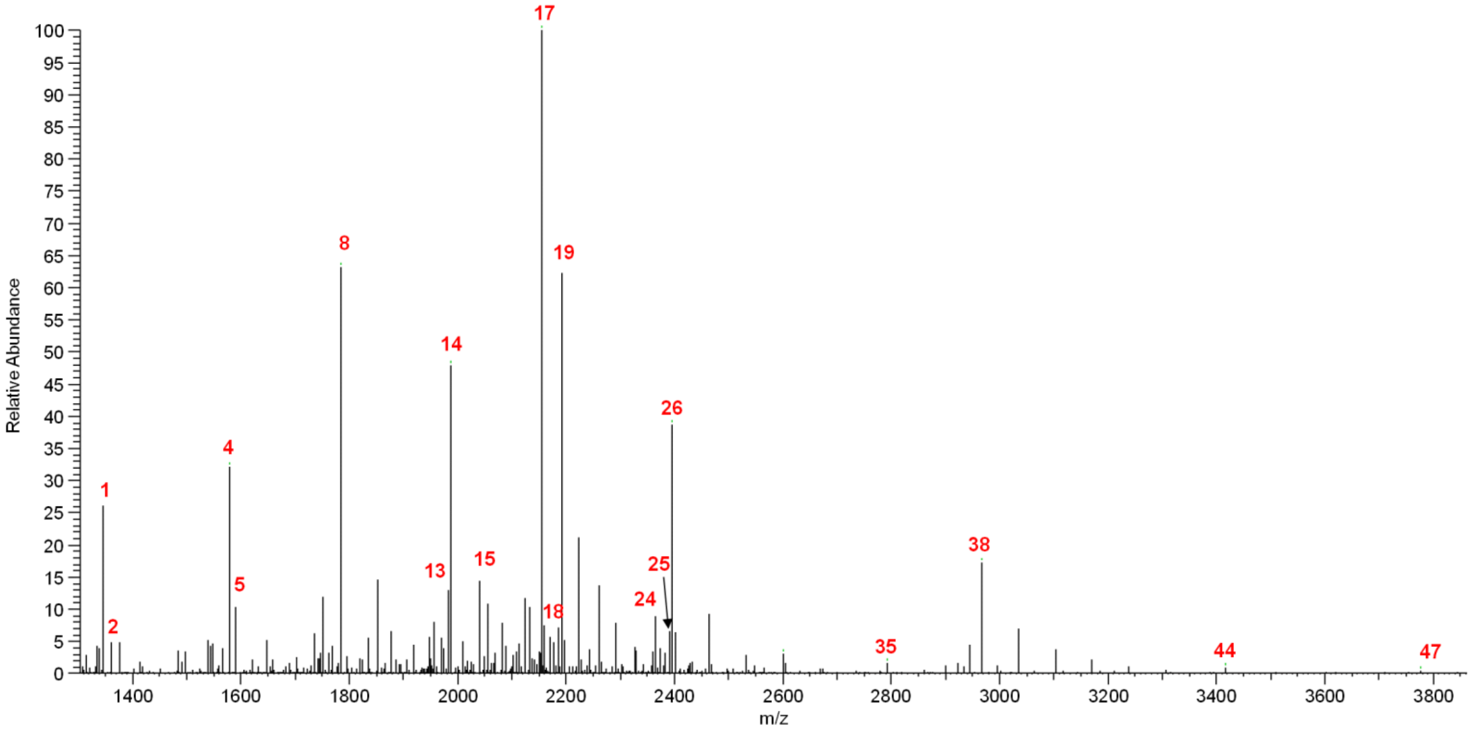

Supp Figure S3: Averaged and deconvoluted LC-ESI-MS spectrum of N-glycans released from NB_1(-*Mgat2)*. N-glycans are labelled with numbers corresponding to Supp Table S3.

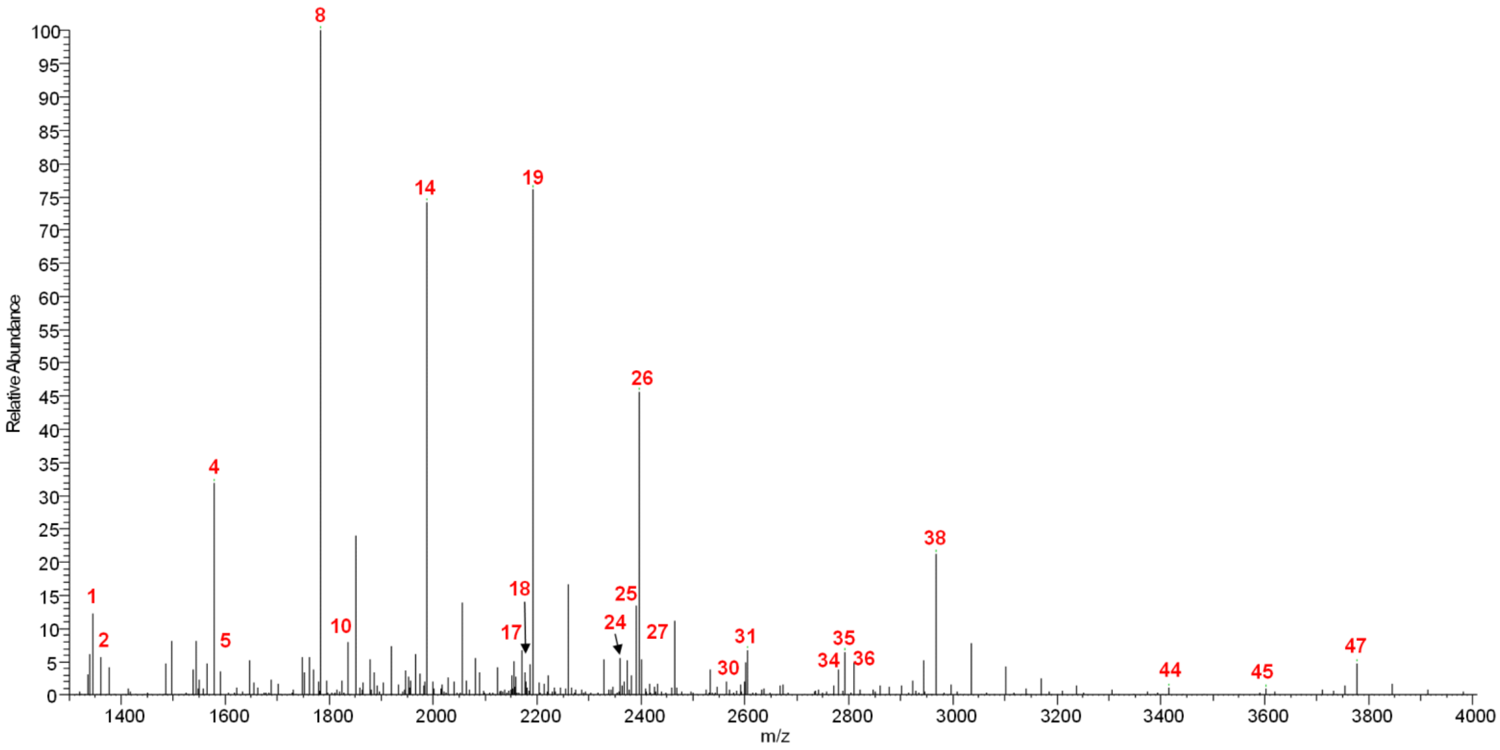

Supp Figure S4: Averaged and deconvoluted LC-ESI-MS spectrum of N-glycans released from NB_1(-*Mgat3*). N-glycans are labelled with numbers corresponding to Supp Table S4.

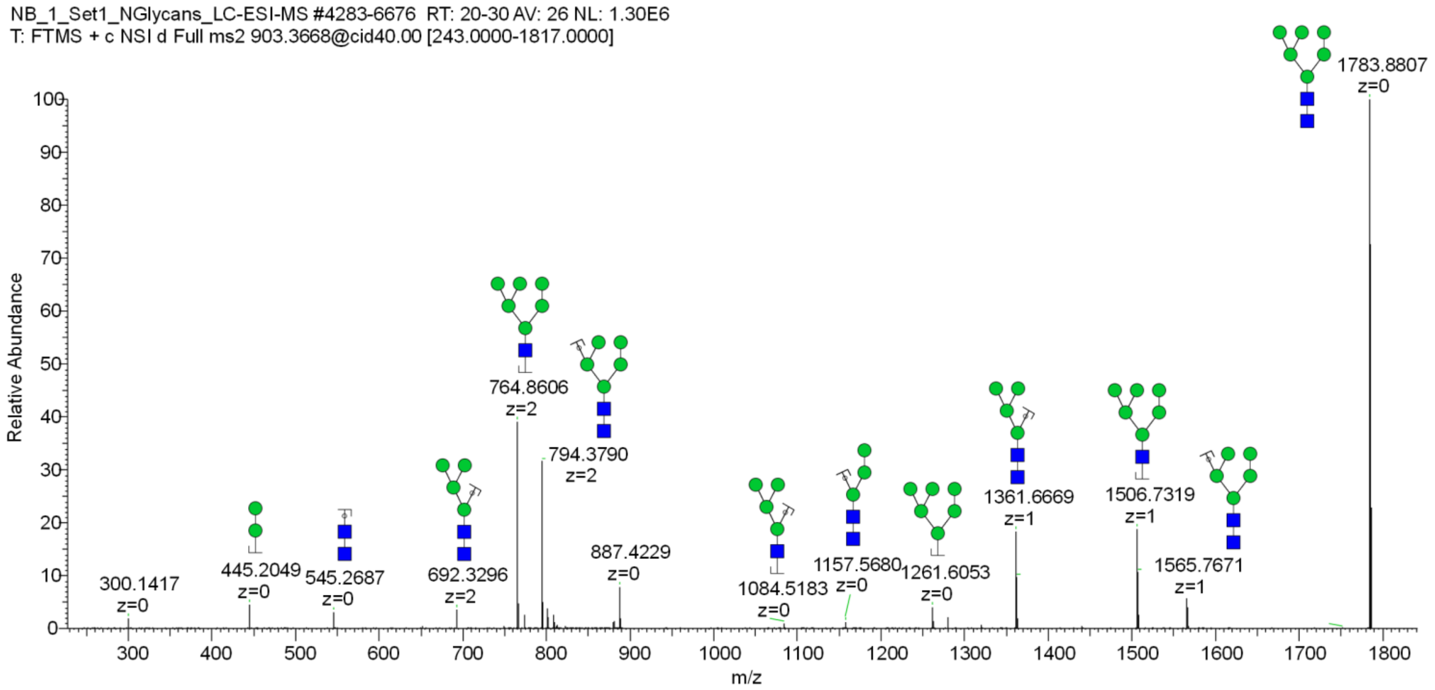

Supp Figure S5: Representative ESI-MS^2 spectrum of N-glycans released from NB_1 with mass m/z 1783.9 showing the glycan fragments which confirms the structure.

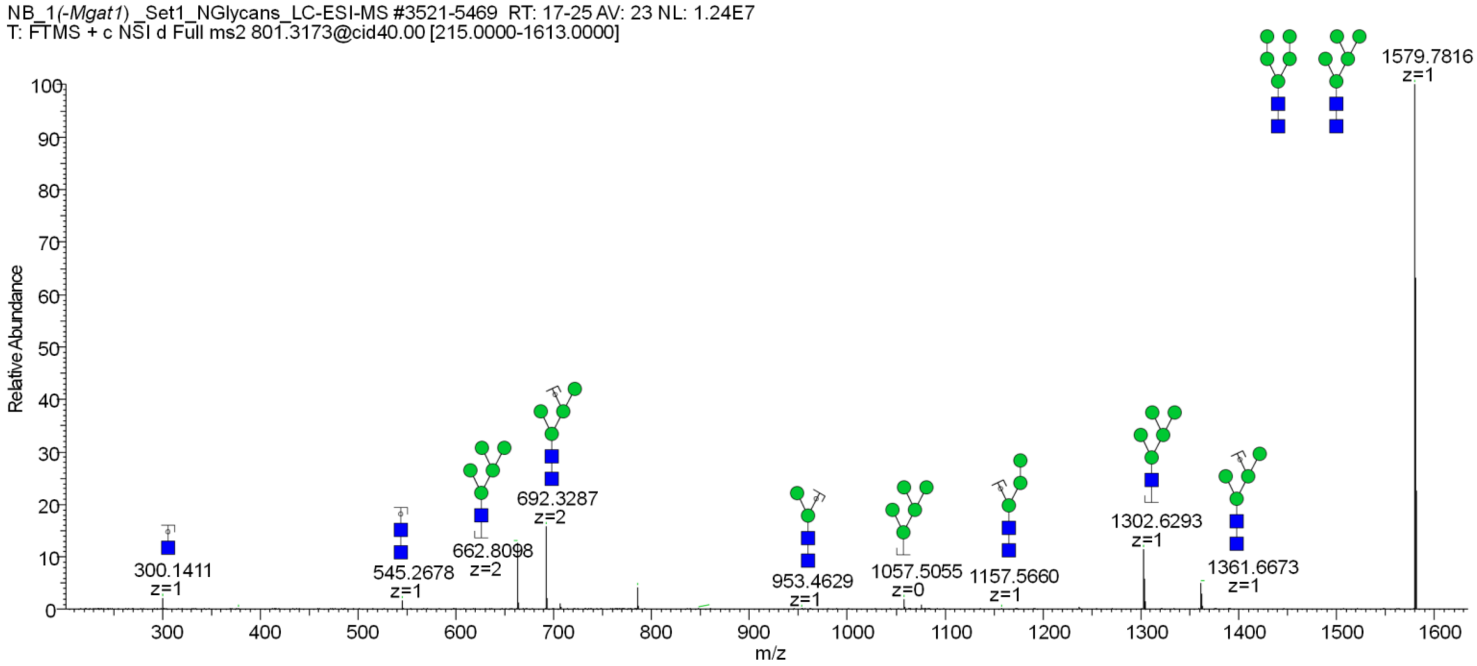

Supp Figure S6: Representative ESI-MS^2 spectrum of N-glycans released from NB_1(-*Mgat1)* with mass m/z 1579.8 showing the glycan fragments which confirms the structure.

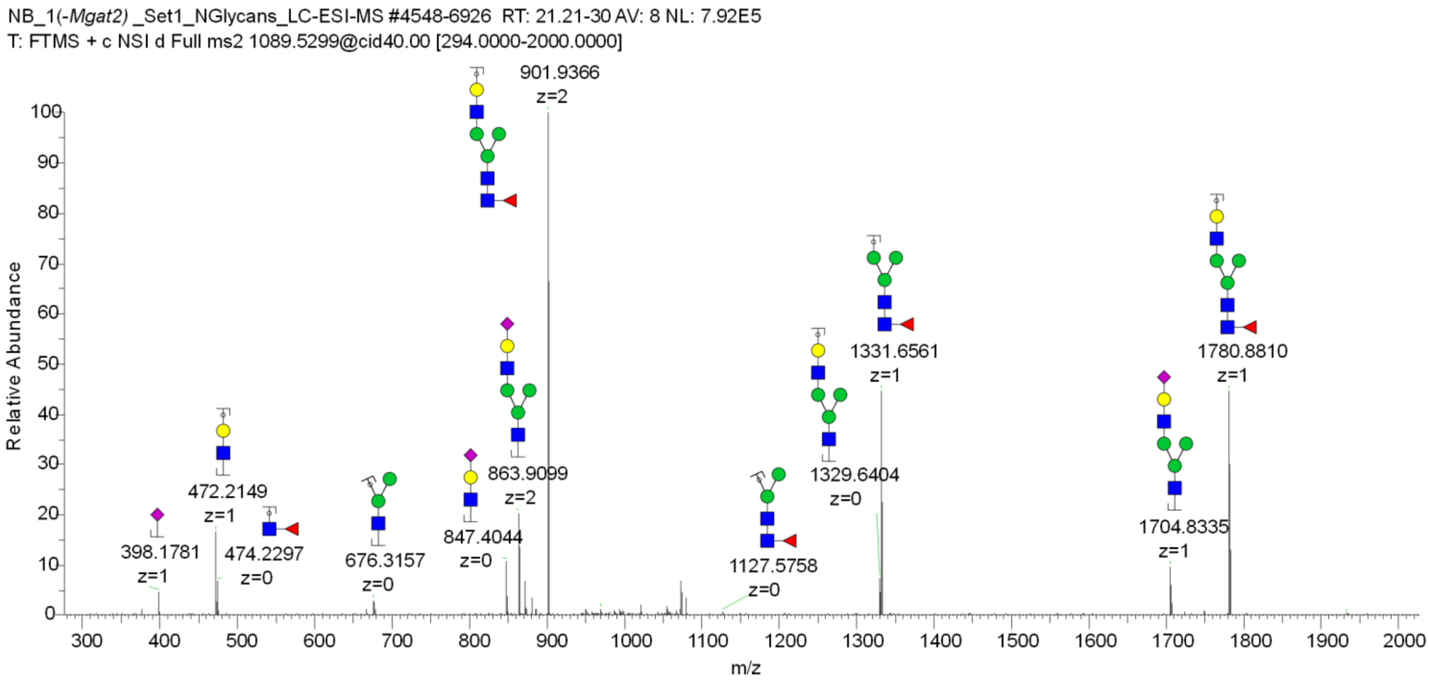

Supp Figure S7: Representative ESI-MS[2] spectrum of N-glycans released from NB_1(-*Mgat2)* with mass m/z 2156.1 showing the glycan fragments which confirms the structure.

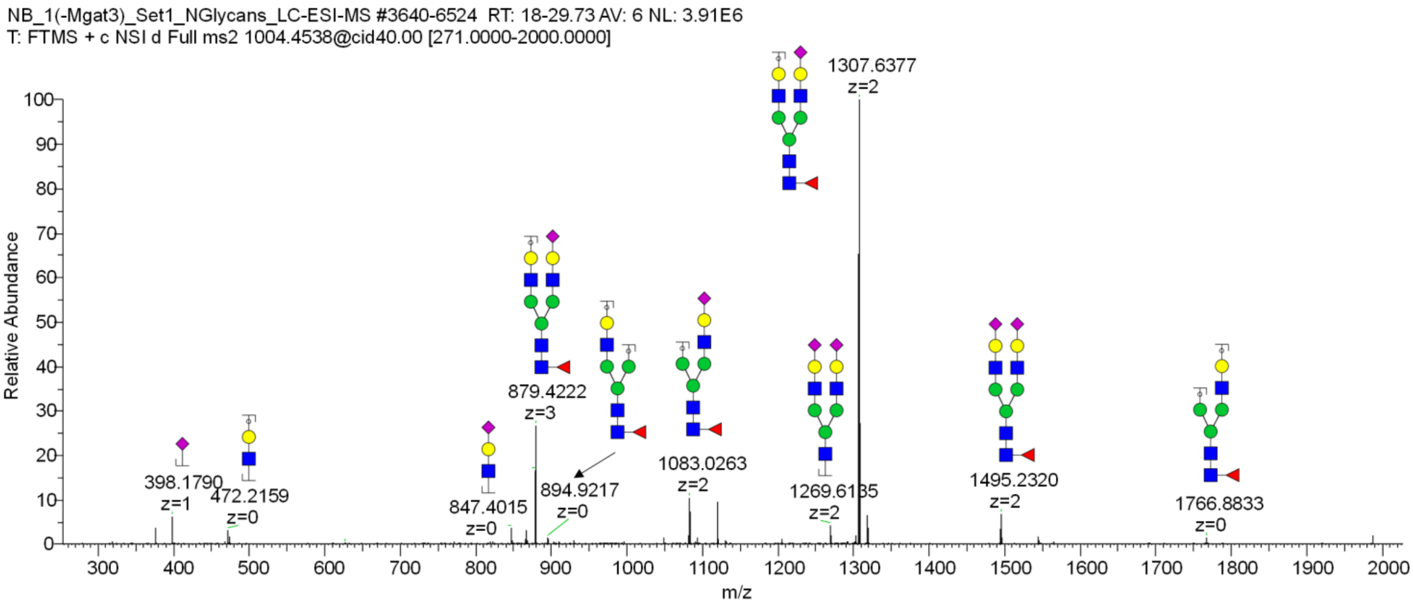

Supp Figure S8: Representative ESI-MS^2 spectrum of N-glycans released from NB_1(-*Mgat3)* with mass m/z 2156.1 showing the glycan fragments which confirms the structure.
