# Supplementary material for: Limited N-Glycan Processing Impacts Chaperone Expression Patterns, Cell Growth and Cell Invasiveness in Neuroblastoma
Source: Biology (Basel). 2023 Feb 11;12(2):293. doi: 10.3390/biology12020293 (PMC9953357; doi:10.3390/biology12020293)
Supplement: Supplementary file 1 [file biology-12-00293-s001.zip › biology-2186381-WB.pdf]

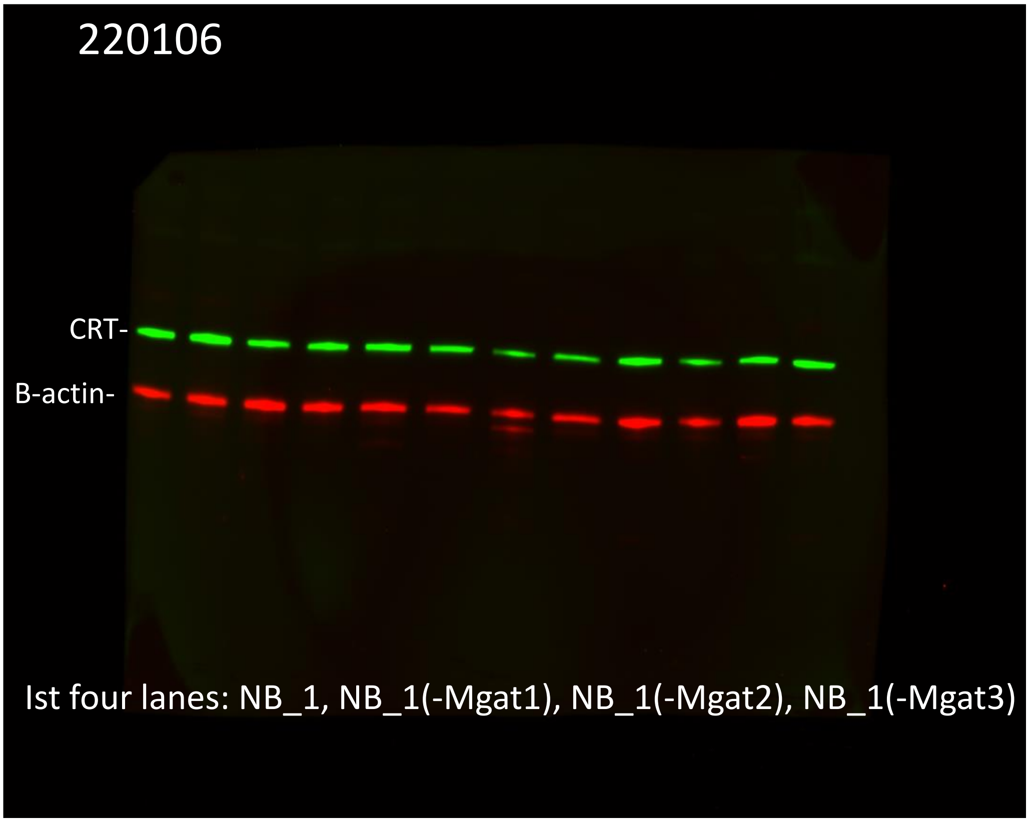
220106
CRT-
B-actin-
Ist four lanes: NB_1, NB_1(-Mgat1), NB_1(-Mgat2), NB_1(-Mgat3)

211215

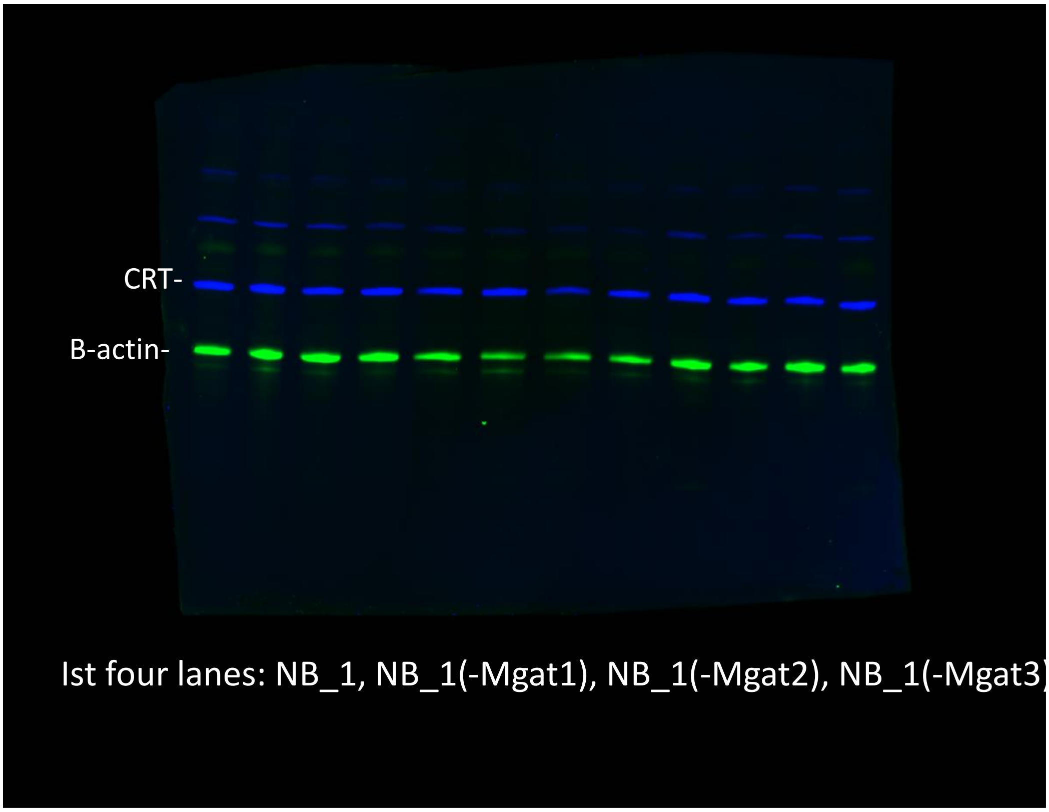
CRT-
B-actin-
Ist four lanes: NB_1, NB_1(-Mgat1), NB_1(-Mgat2), NB_1(-Mgat3)

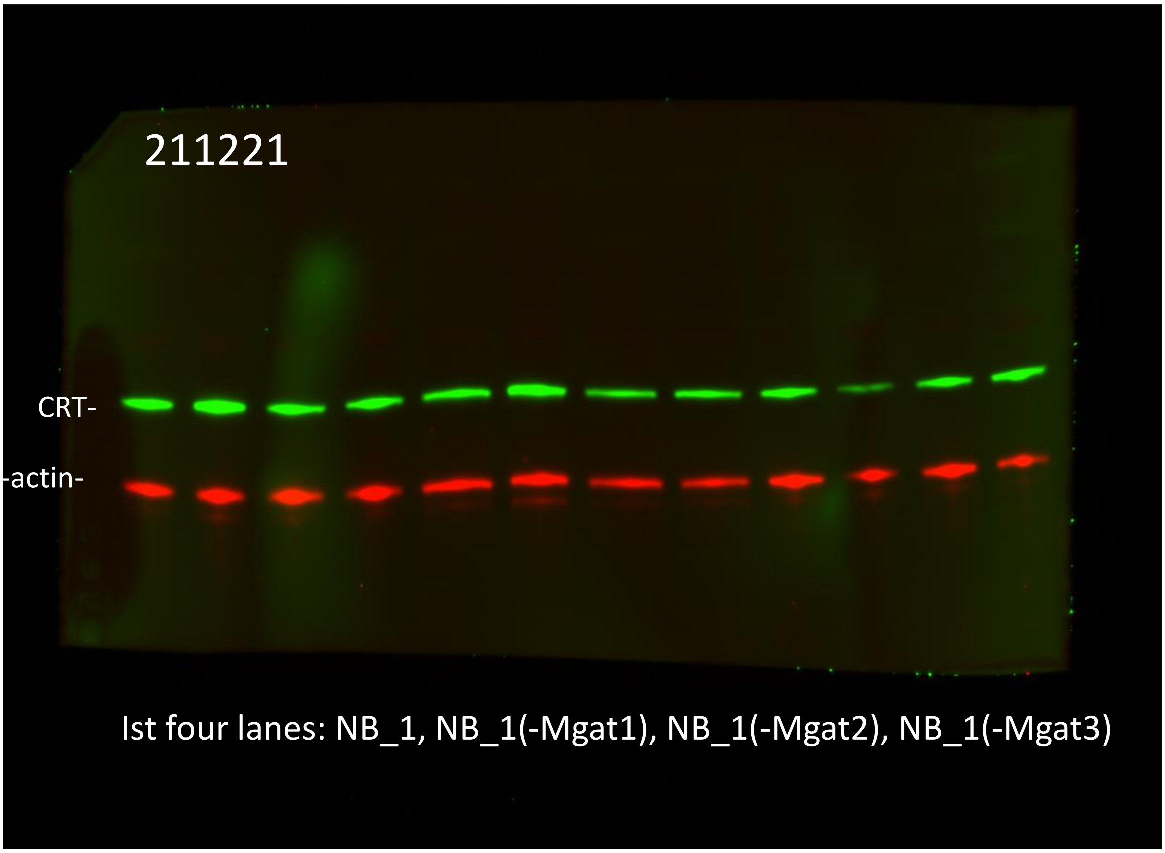
211221
CRT-
-actin-
Ist four lanes: NB_1, NB_1(-Mgat1), NB_1(-Mgat2), NB_1(-Mgat3)

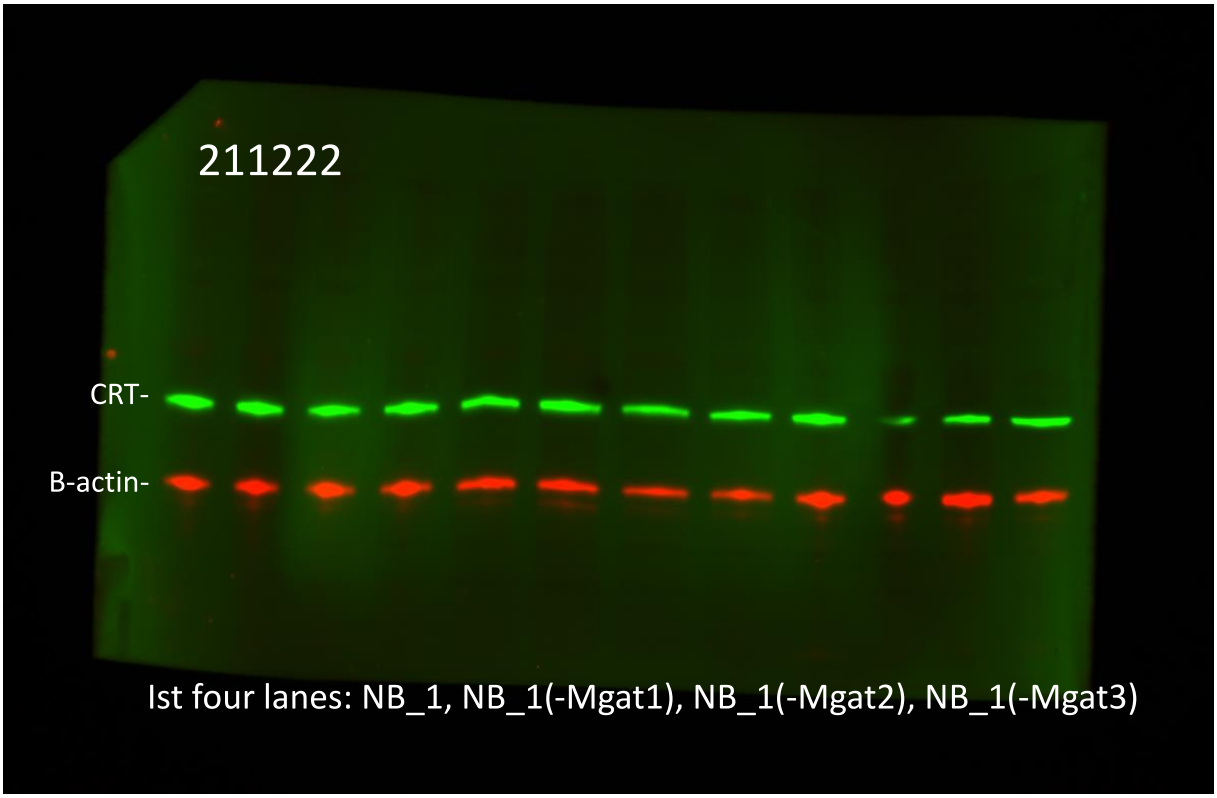
211222
CRT-
B-actin-
Ist four lanes: NB_1, NB_1(-Mgat1), NB_1(-Mgat2), NB_1(-Mgat3)

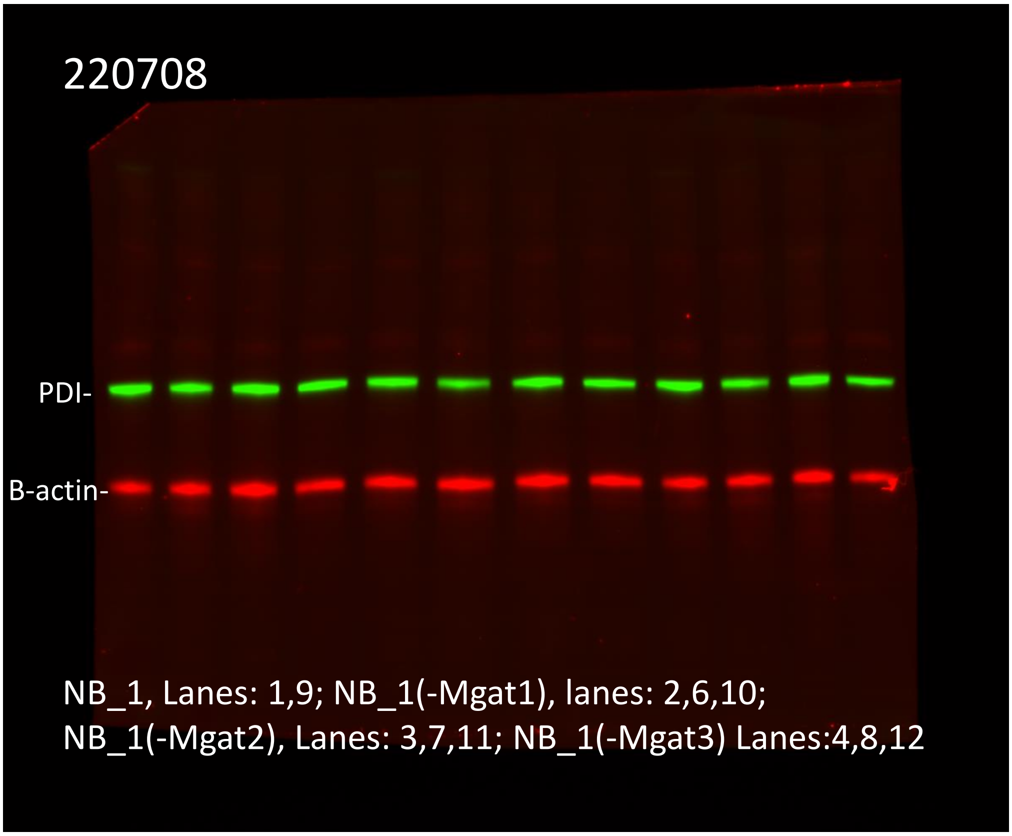
220708
PDI-
B-actin-
NB_1, Lanes: 1,9; NB_1(-Mgat1), lanes: 2,6,10;
NB_1(-Mgat2), Lanes: 3,7,11; NB_1(-Mgat3) Lanes:4,8,12

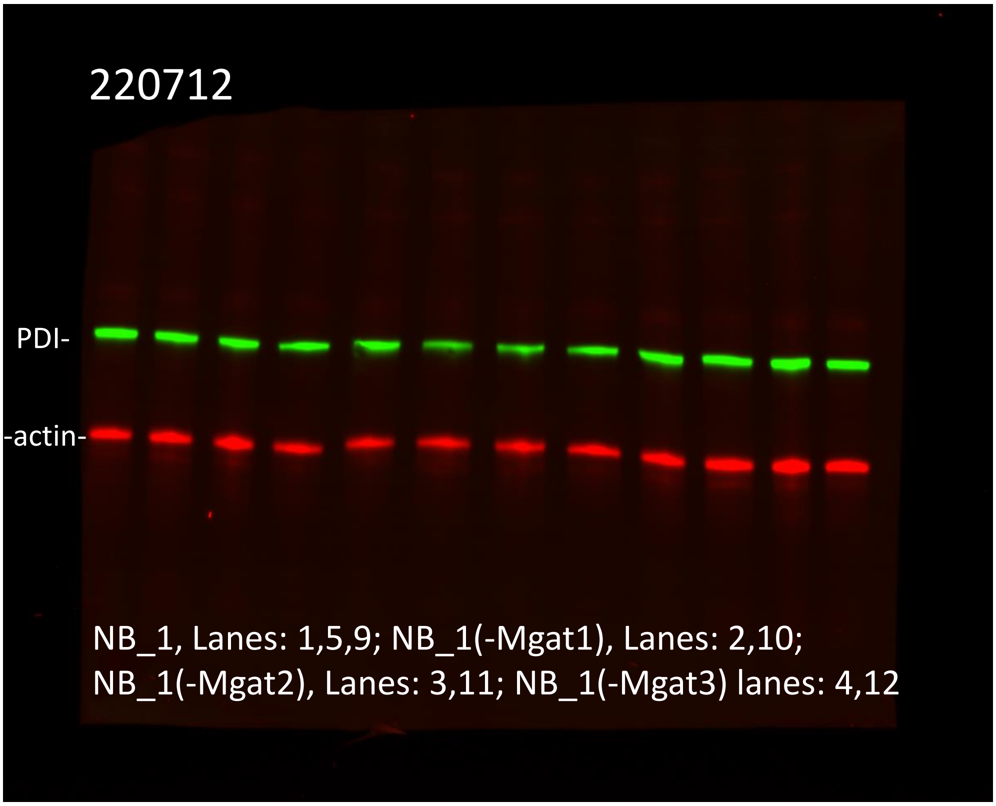
220712
PDI-
-actin-
NB_1, Lanes: 1,5,9; NB_1(-Mgat1), Lanes: 2,10;
NB_1(-Mgat2), Lanes: 3,11; NB_1(-Mgat3) lanes: 4,12

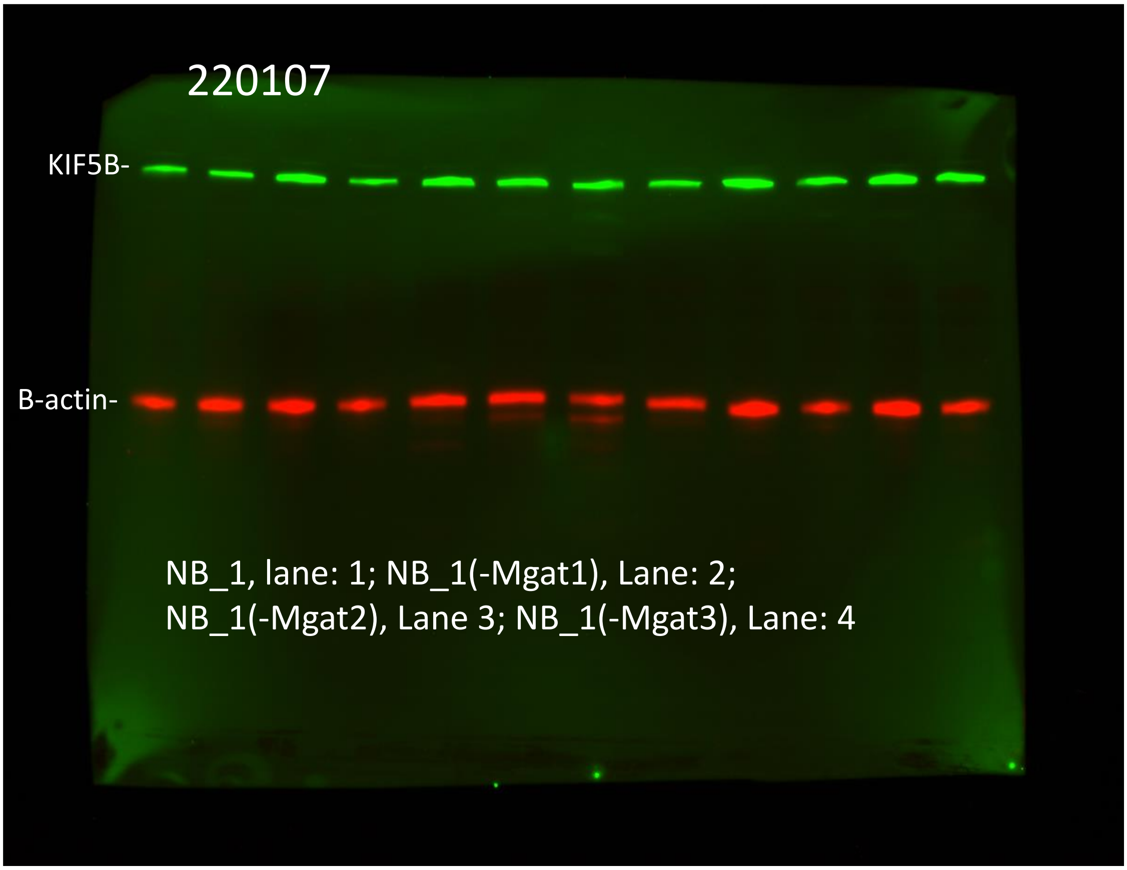
220107
KIF5B-
B-actin-
NB_1, lane: 1; NB_1(-Mgat1), Lane: 2;
NB_1(-Mgat2), Lane 3; NB_1(-Mgat3), Lane: 4

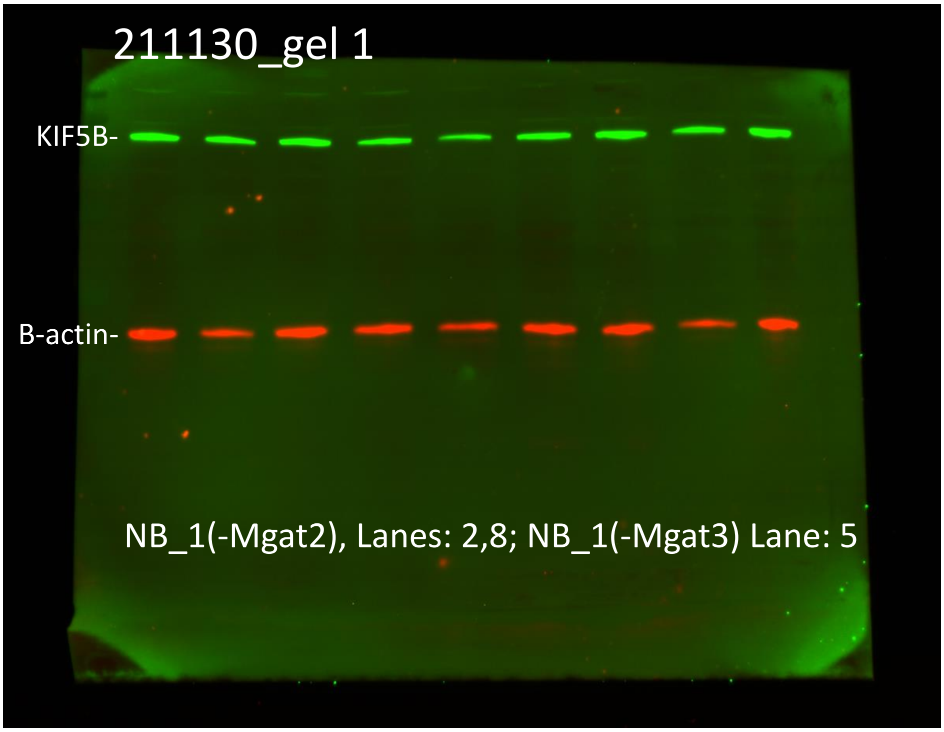
211130_gel 1
KIF5B-
B-actin-
NB_1(-Mgat2), Lanes: 2,8; NB_1(-Mgat3) Lane: 5

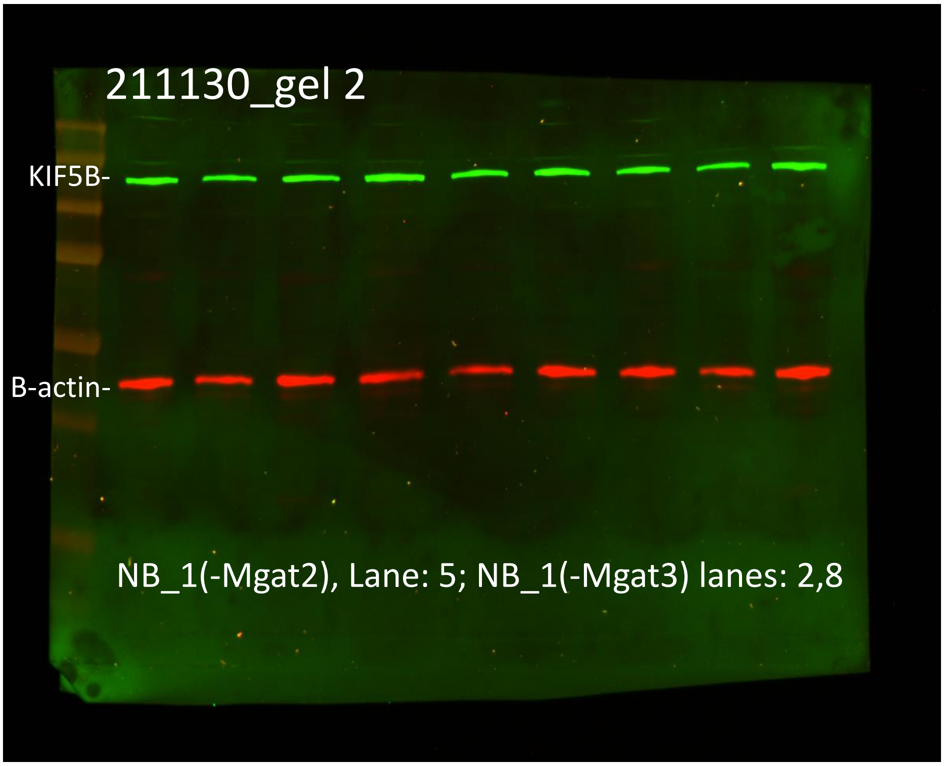
211130_gel 2
KIF5B-
B-actin-
NB_1(-Mgat2), Lane: 5; NB_1(-Mgat3) lanes: 2,8

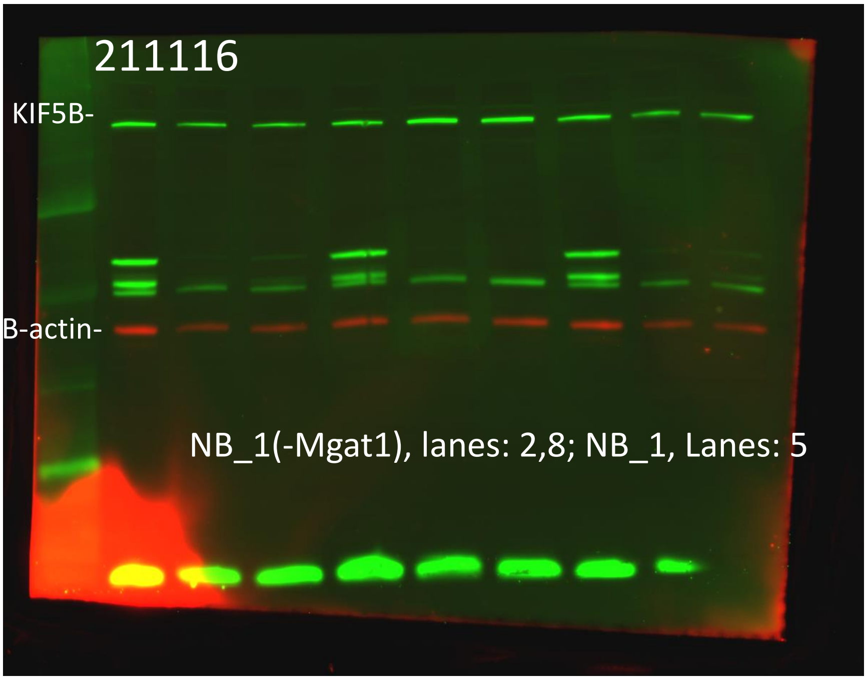
211116
KIF5B-
B-actin-
NB_1(-Mgat1), lanes: 2,8; NB_1, Lanes: 5

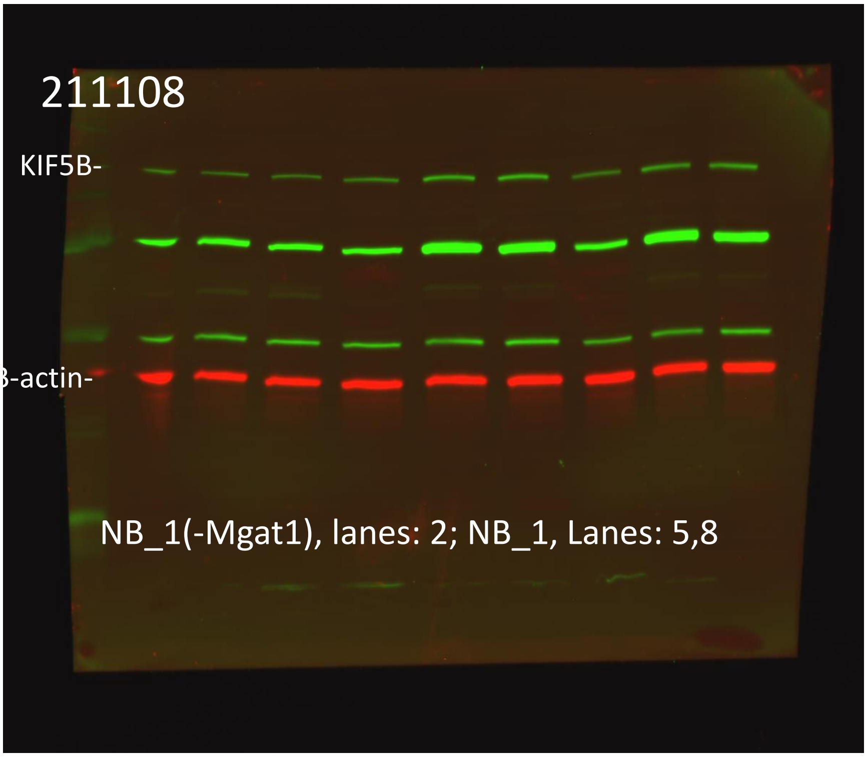
211108
KIF5B-
-actin-
NB_1(-Mgat1), lanes: 2; NB_1, Lanes: 5,8

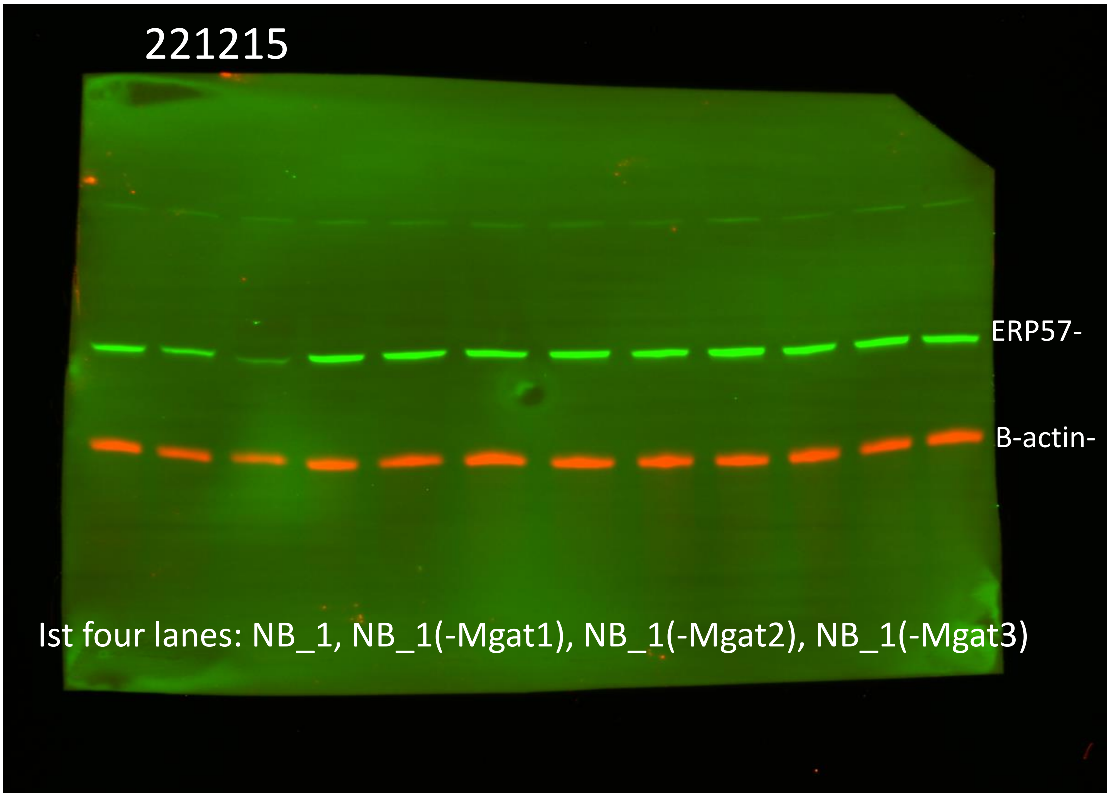
221215
ERP57-
B-actin-
Ist four lanes: NB_1, NB_1(-Mgat1), NB_1(-Mgat2), NB_1(-Mgat3)

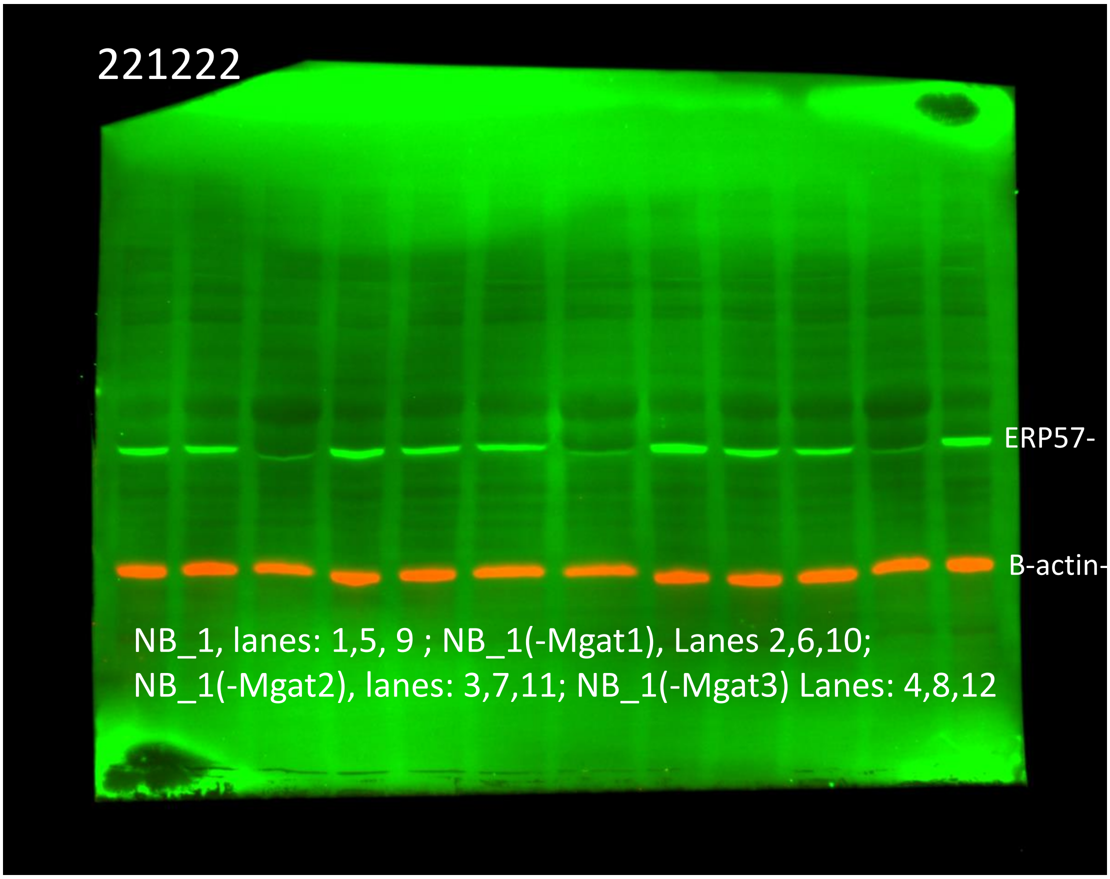
221222
ERP57-
B-actin-
NB_1, lanes: 1,5, 9 ; NB_1(-Mgat1), Lanes 2,6,10;
NB_1(-Mgat2), lanes: 3,7,11; NB_1(-Mgat3) Lanes: 4,8,12

220110

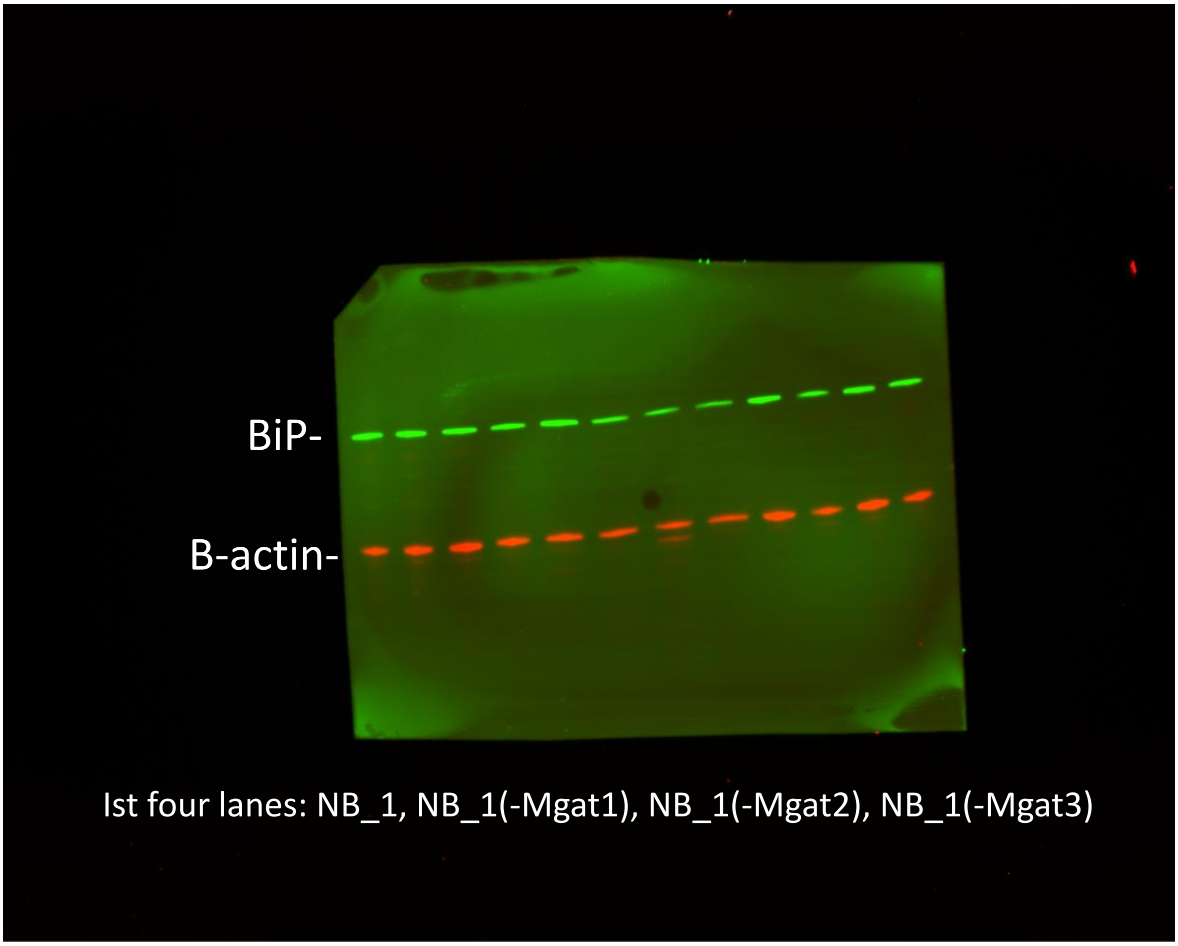

220110

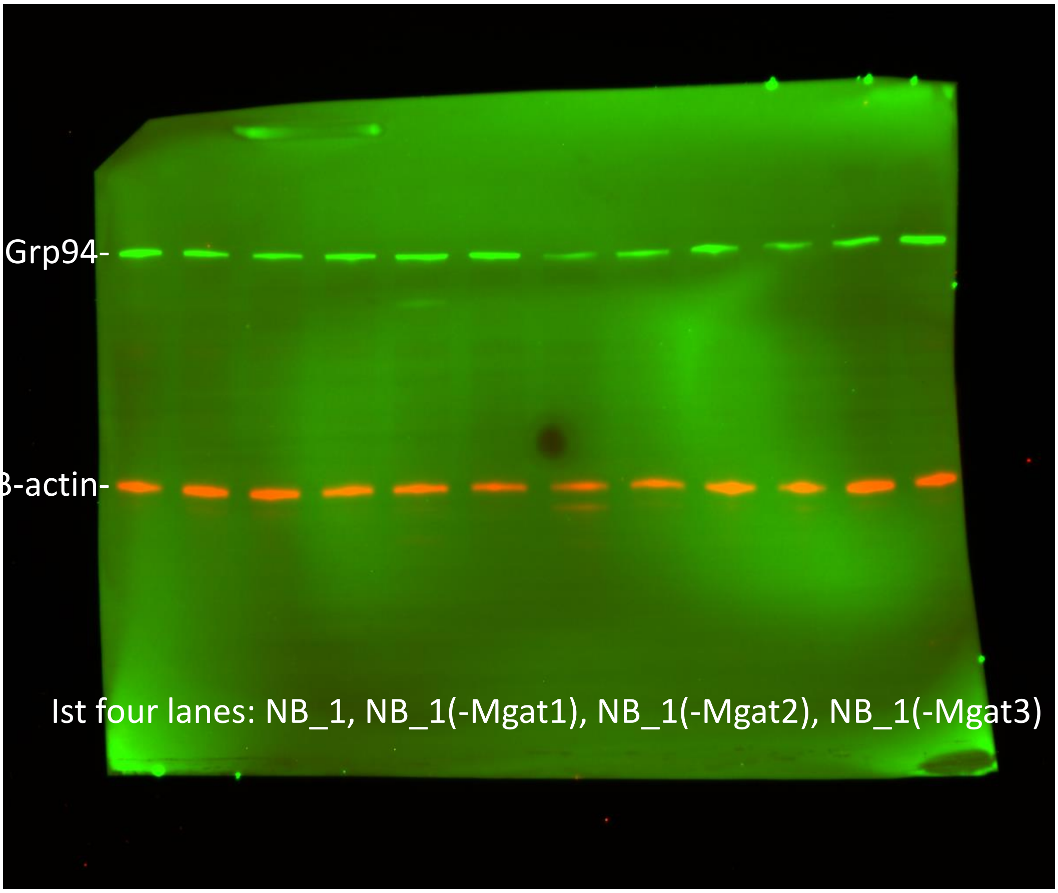

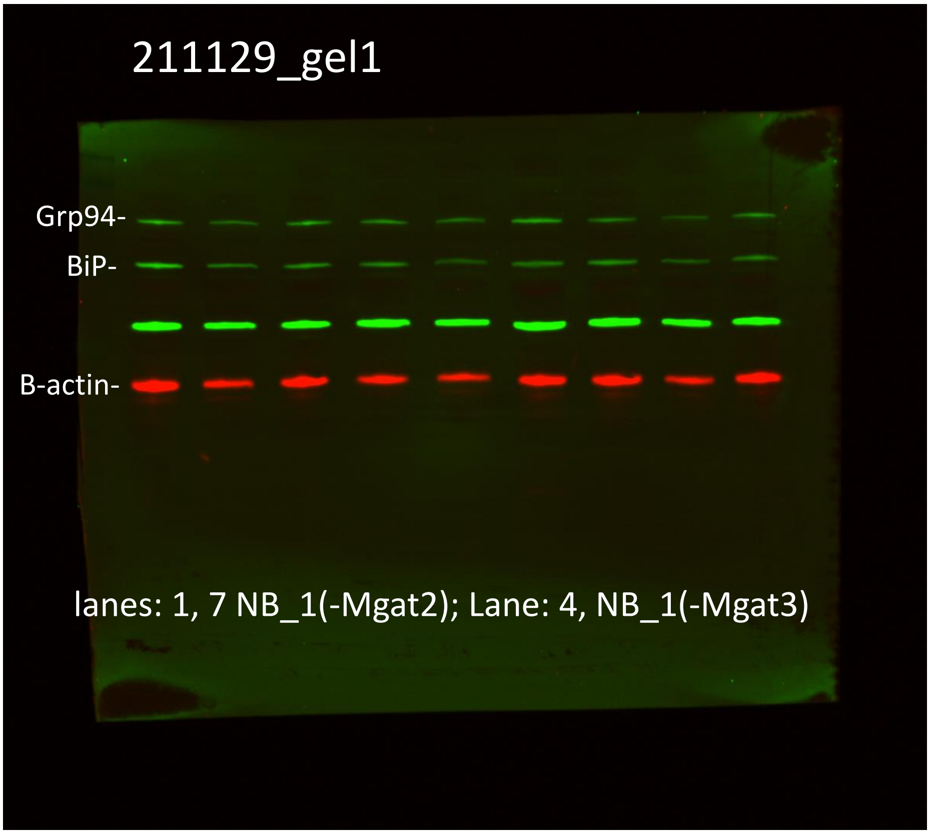
211129_gel1
Grp94-
BiP-
B-actin-
lanes: 1, 7 NB_1(-Mgat2); Lane: 4, NB_1(-Mgat3)

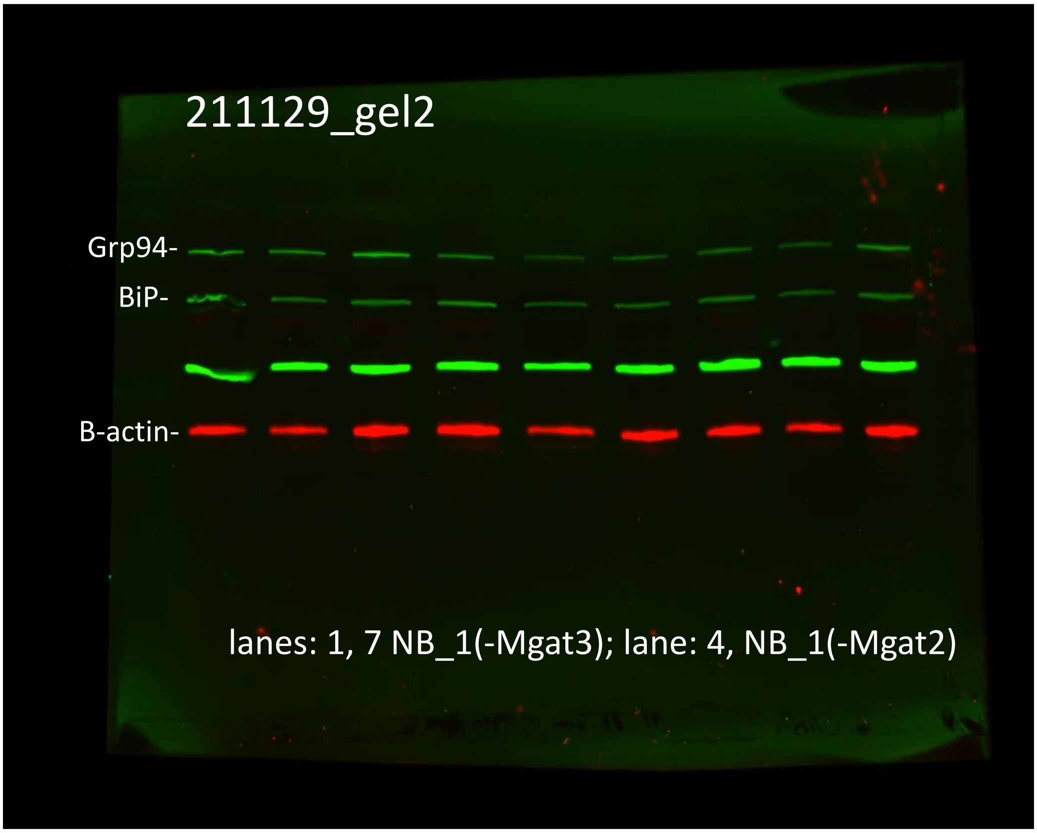
211129_gel2
Grp94-
BiP-
B-actin-
lanes: 1, 7 NB_1(-Mgat3); lane: 4, NB_1(-Mgat2)

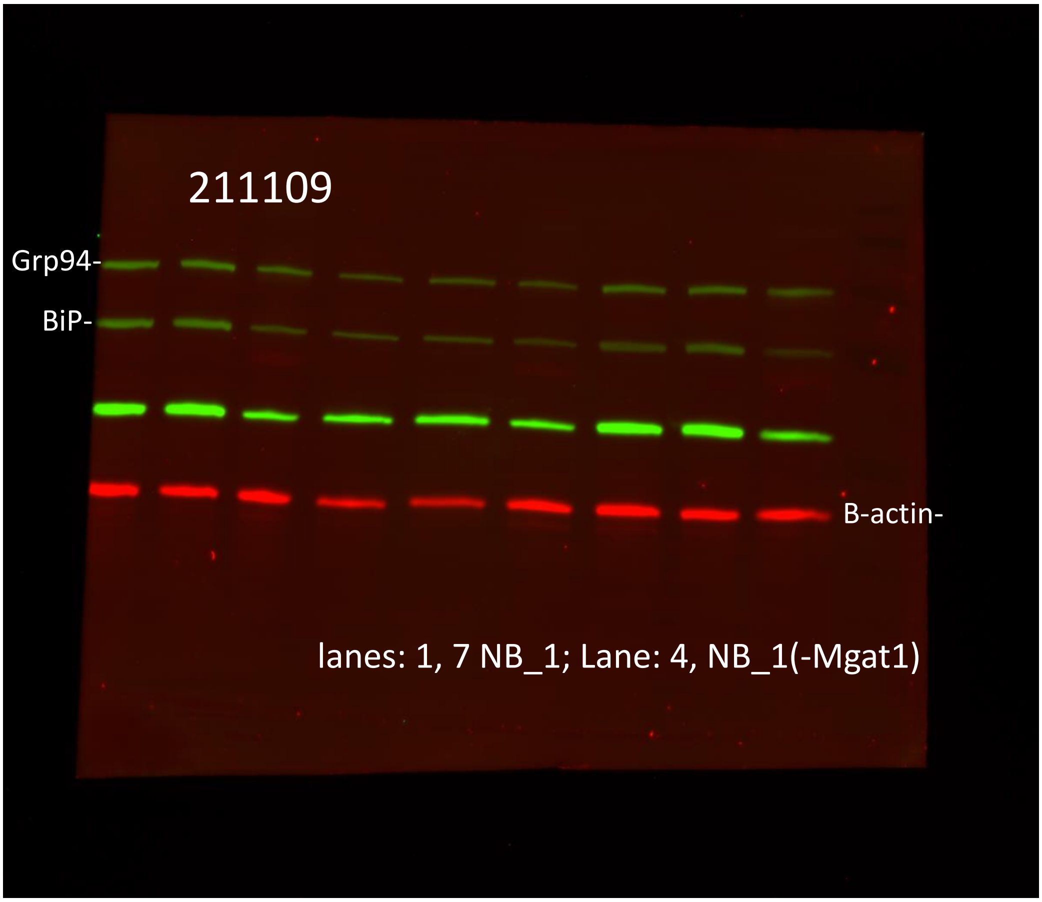
211109
Grp94-
BiP-
B-actin-
lanes: 1, 7 NB_1; Lane: 4, NB_1(-Mgat1)

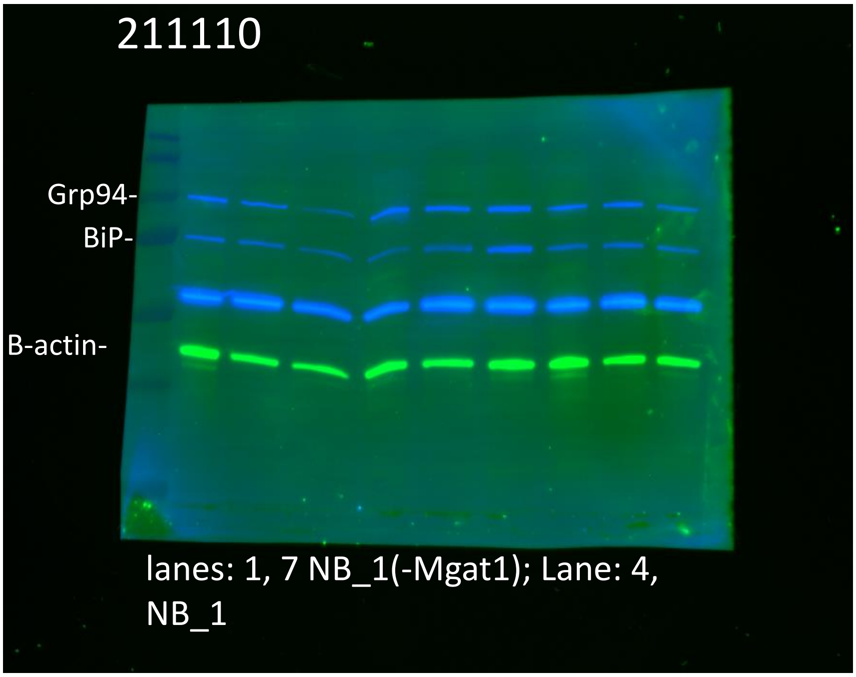
211110
Grp94-
BiP-
B-actin-
lanes: 1, 7 NB_1(-Mgat1); Lane: 4,
NB_1

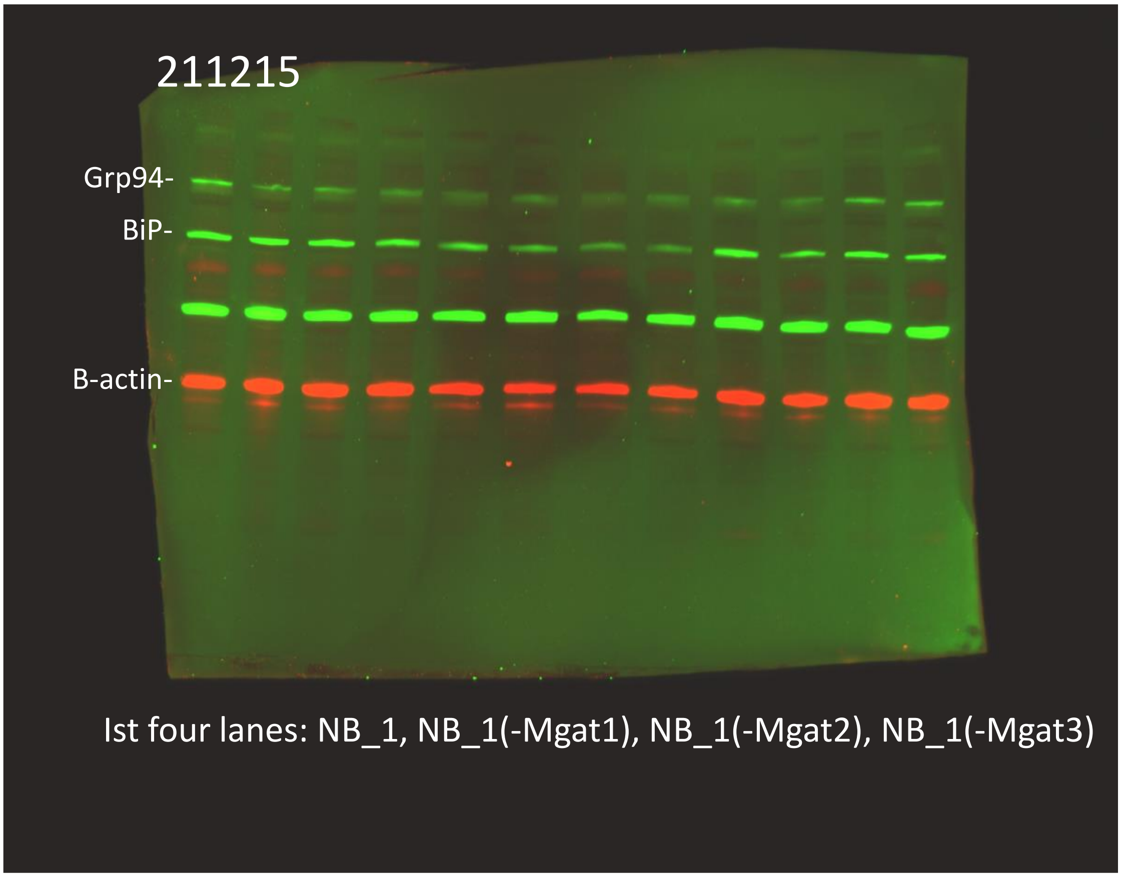
211215
Grp94-
BiP-
B-actin-
Ist four lanes: NB_1, NB_1(-Mgat1), NB_1(-Mgat2), NB_1(-Mgat3)

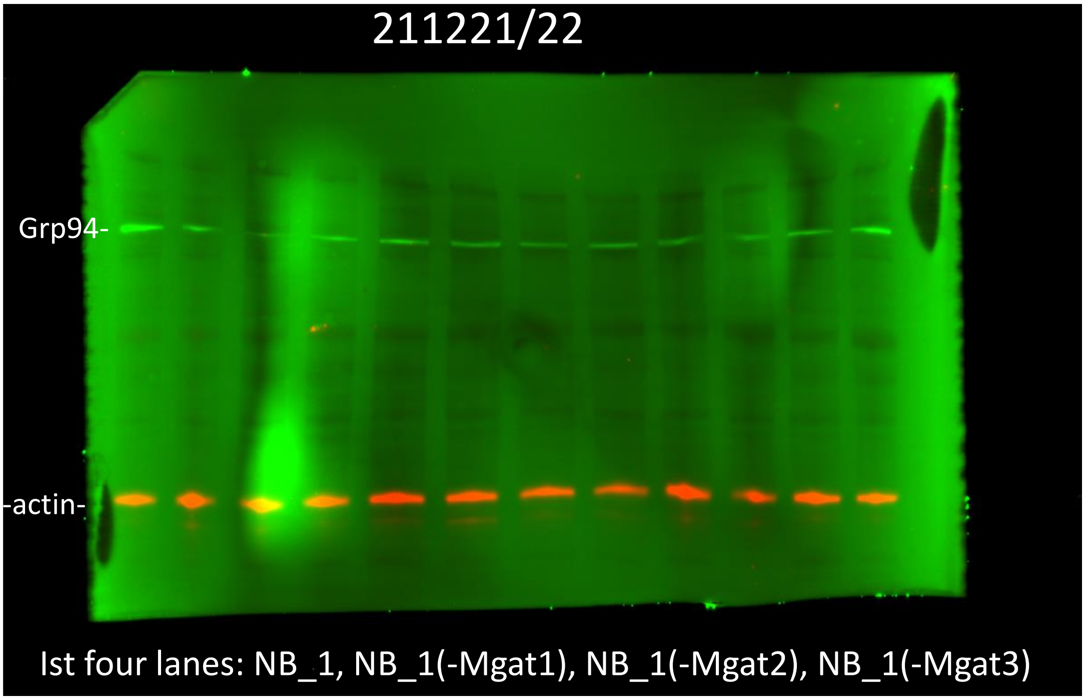
211221/22
Grp94-
-actin-
Ist four lanes: NB_1, NB_1(-Mgat1), NB_1(-Mgat2), NB_1(-Mgat3)

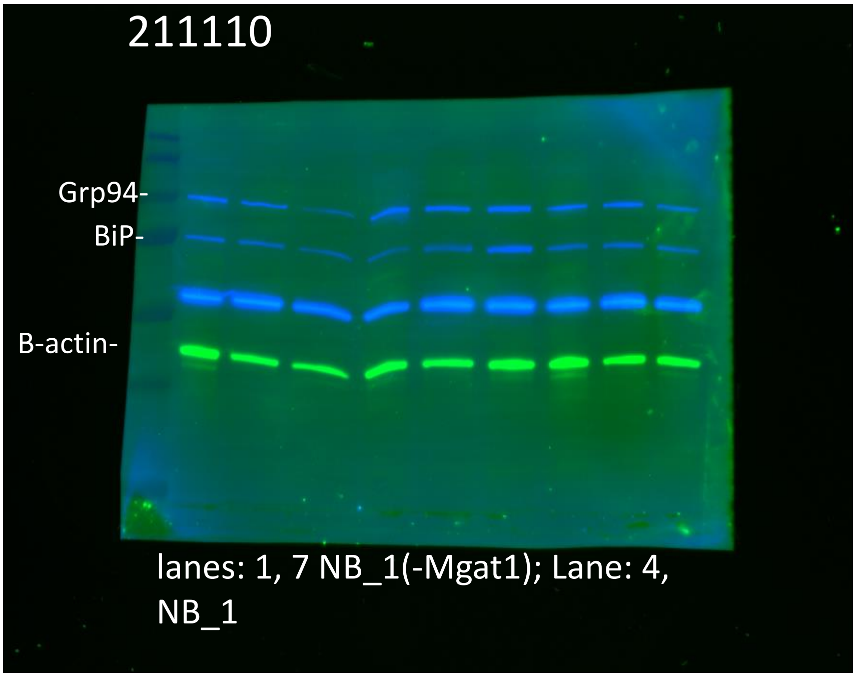
211110
Grp94-
BiP-
B-actin-
lanes: 1, 7 NB_1(-Mgat1); Lane: 4, NB_1

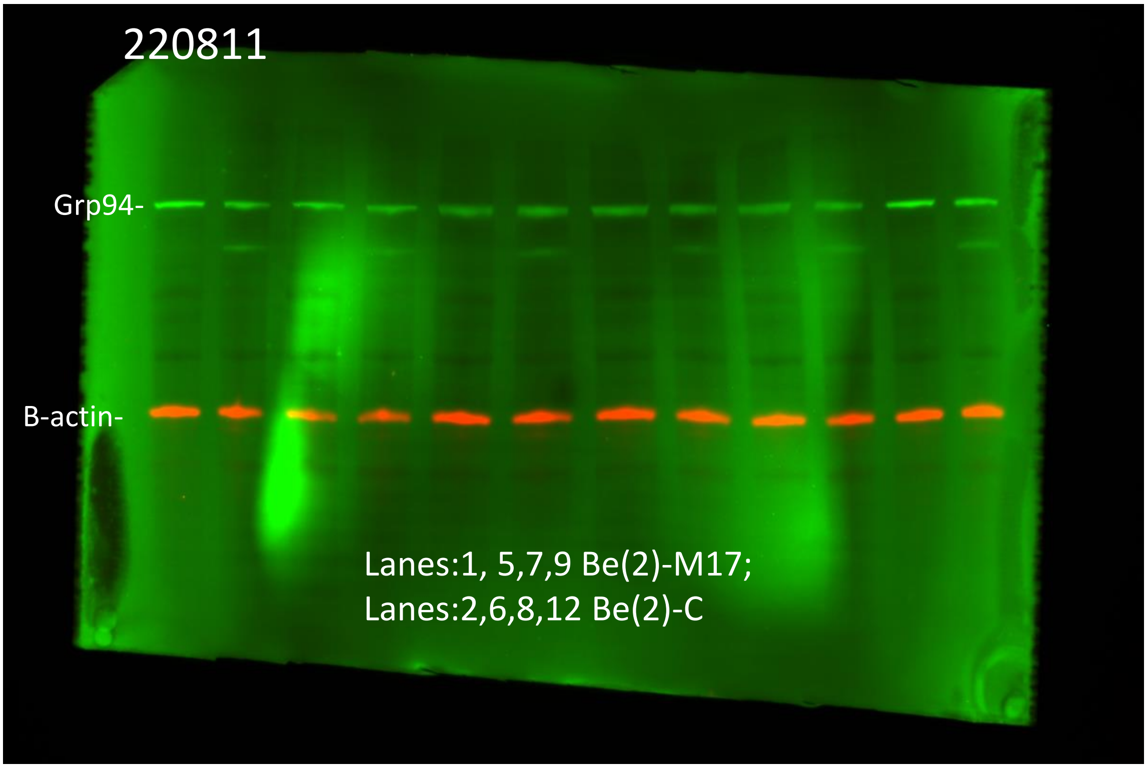
220811
Grp94-
B-actin-
Lanes:1, 5,7,9 Be(2)-M17;
Lanes:2,6,8,12 Be(2)-C

Human

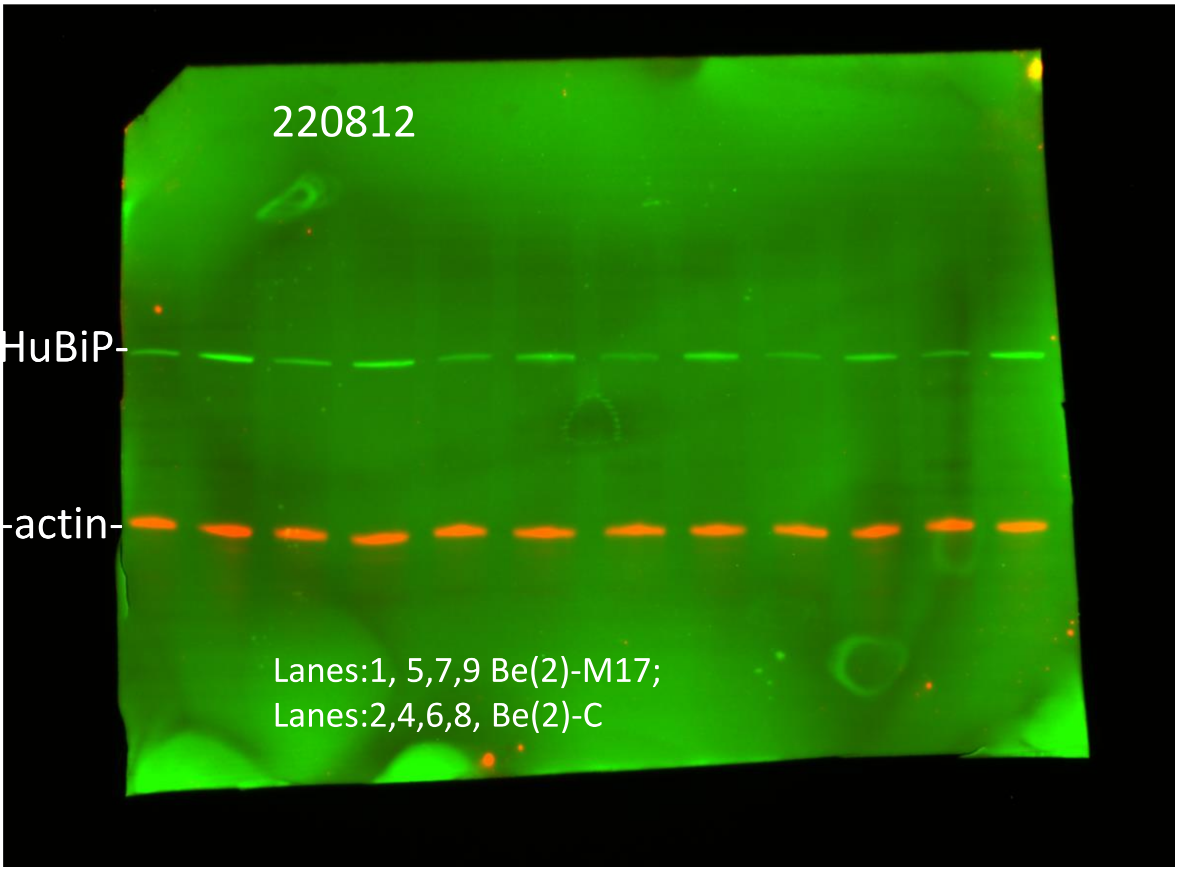
220812
HuBiP-
-actin-
Lanes:1, 5,7,9 Be(2)-M17;
Lanes:2,4,6,8, Be(2)-C

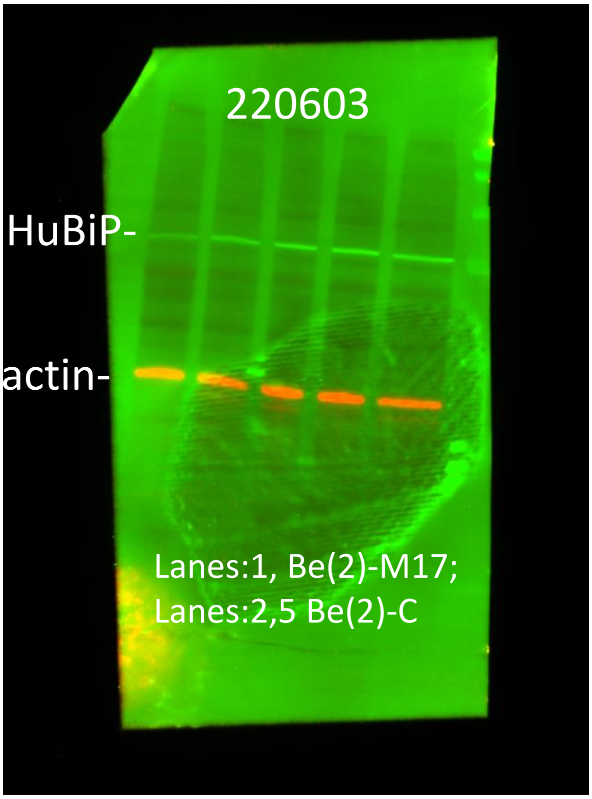
220603
HuBiP-
actin-
Lanes:1, Be(2)-M17;
Lanes:2,5 Be(2)-C

Human

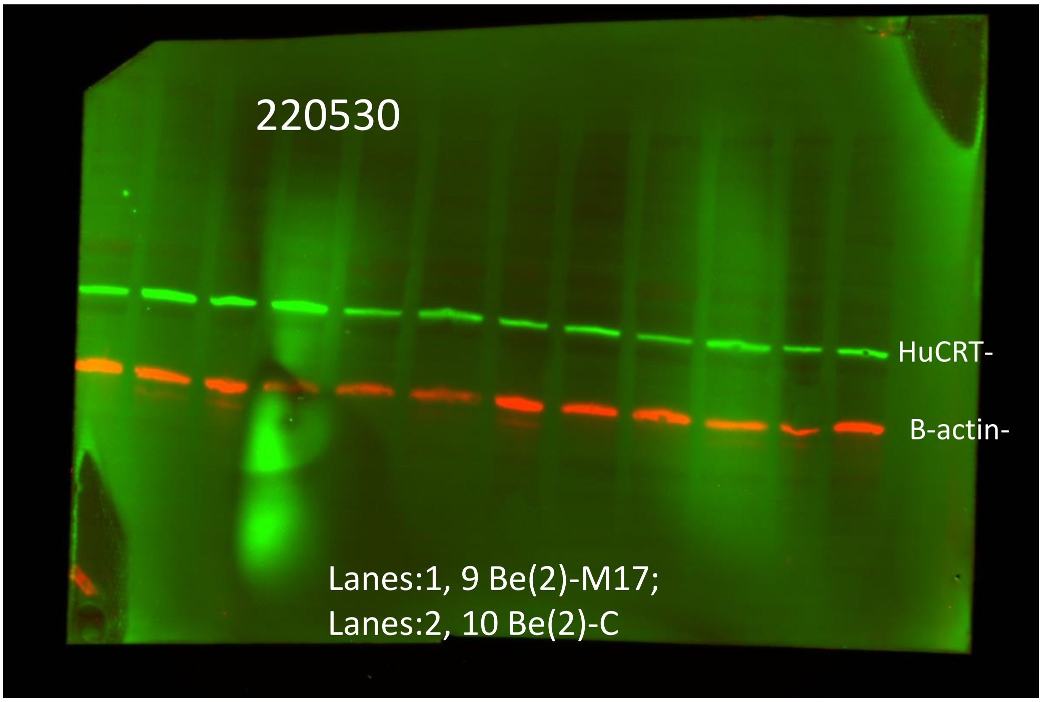
220530
HuCRT-
B-actin-
Lanes:1, 9 Be(2)-M17;
Lanes:2, 10 Be(2)-C

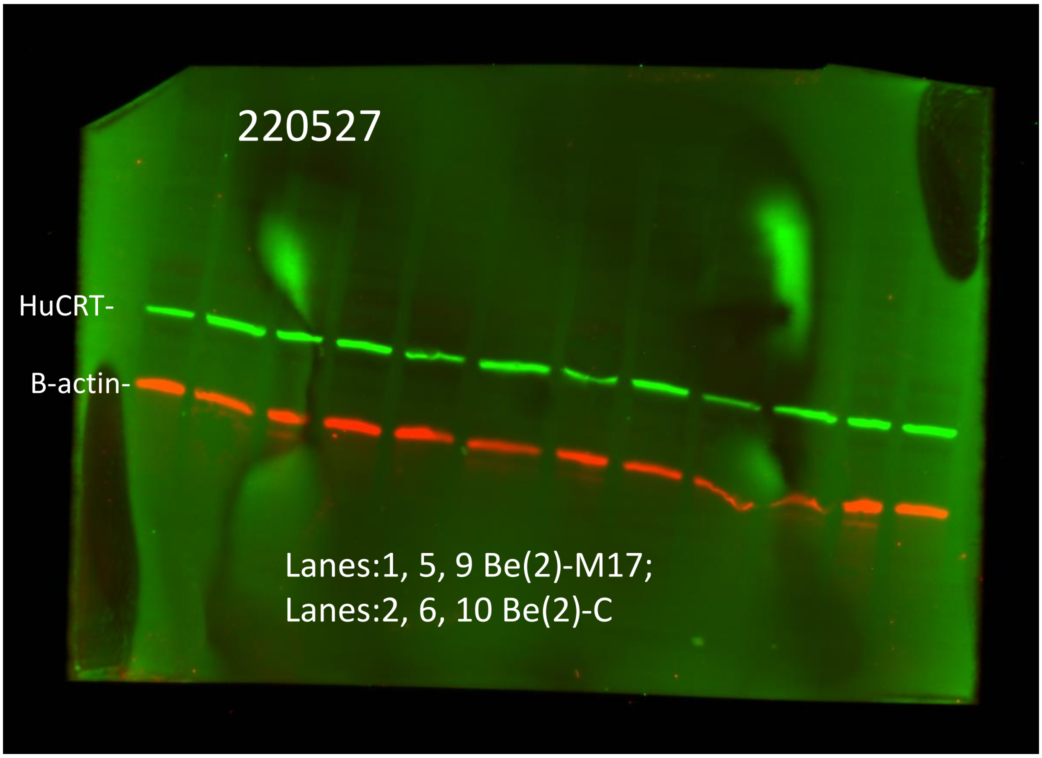
220527
HuCRT-
B-actin-
Lanes:1, 5, 9 Be(2)-M17;
Lanes:2, 6, 10 Be(2)-C

Human

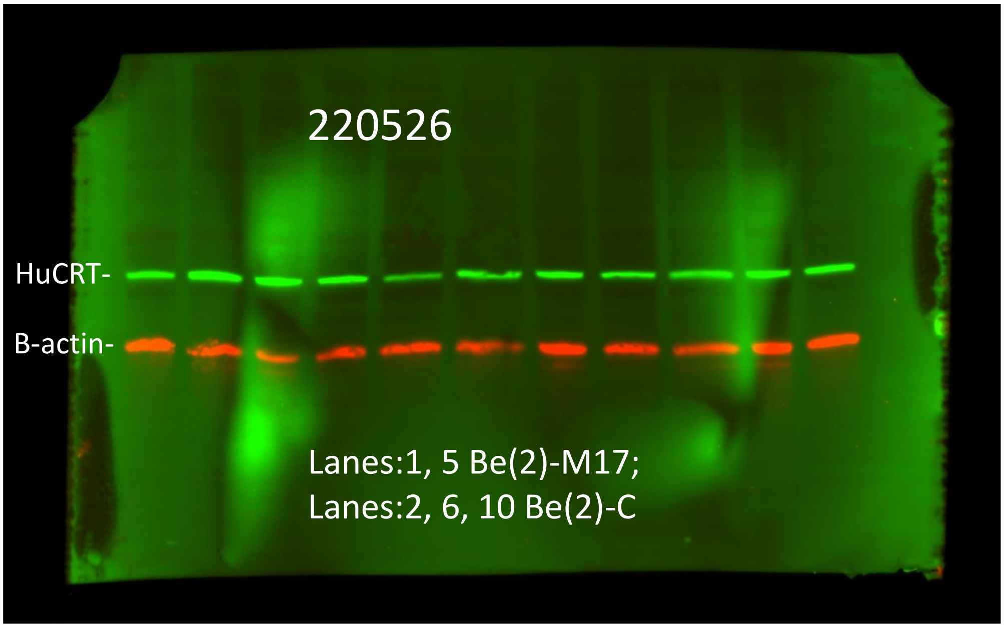
220526
HuCRT-
B-actin-
Lanes:1, 5 Be(2)-M17;
Lanes:2, 6, 10 Be(2)-C

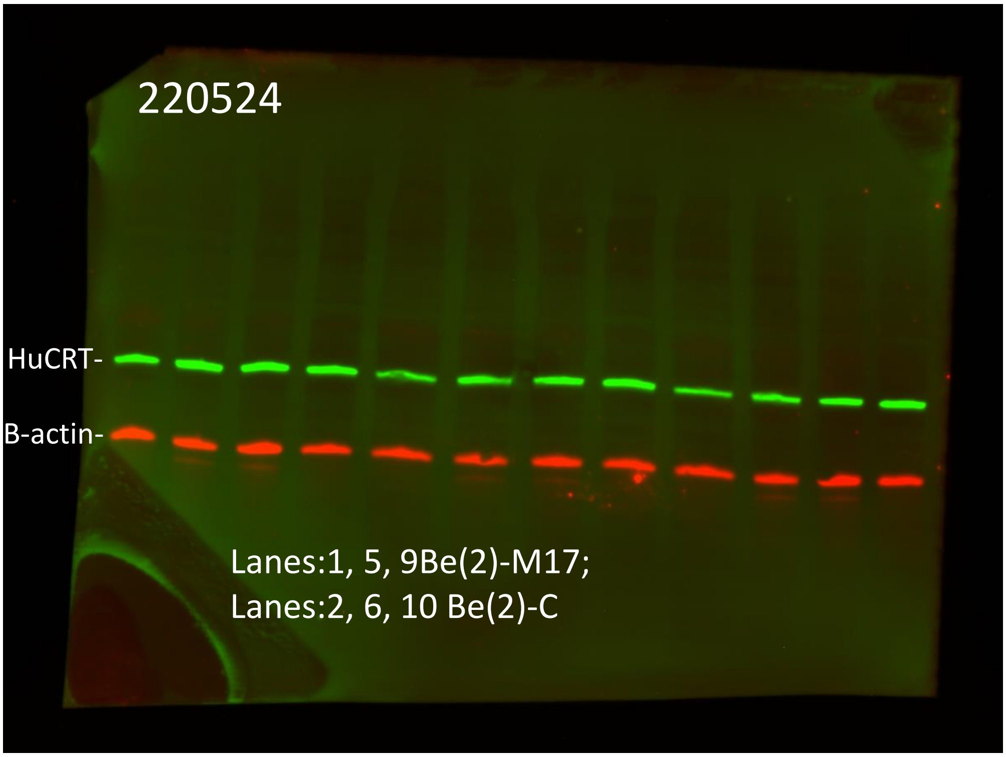
220524
HuCRT-
B-actin-
Lanes:1, 5, 9Be(2)-M17;
Lanes:2, 6, 10 Be(2)-C

Human

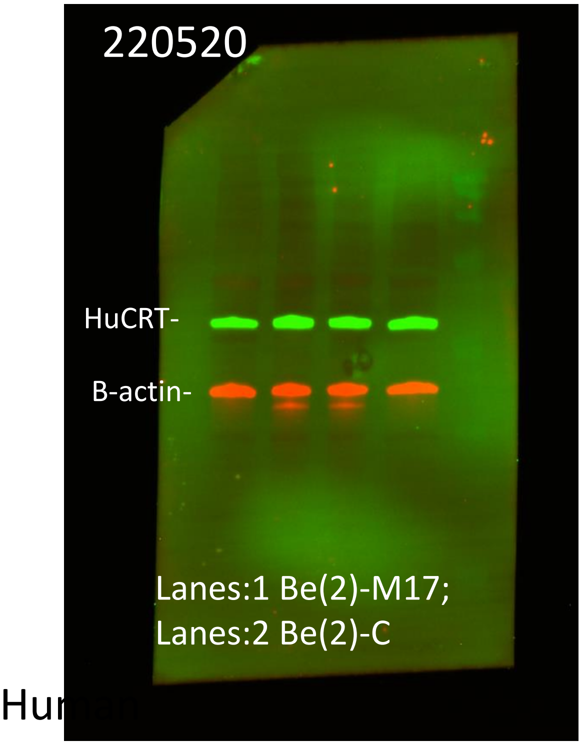
220520
HuCRT-
B-actin-
Lanes:1 Be(2)-M17;
Lanes:2 Be(2)-C

Hu[illegible]

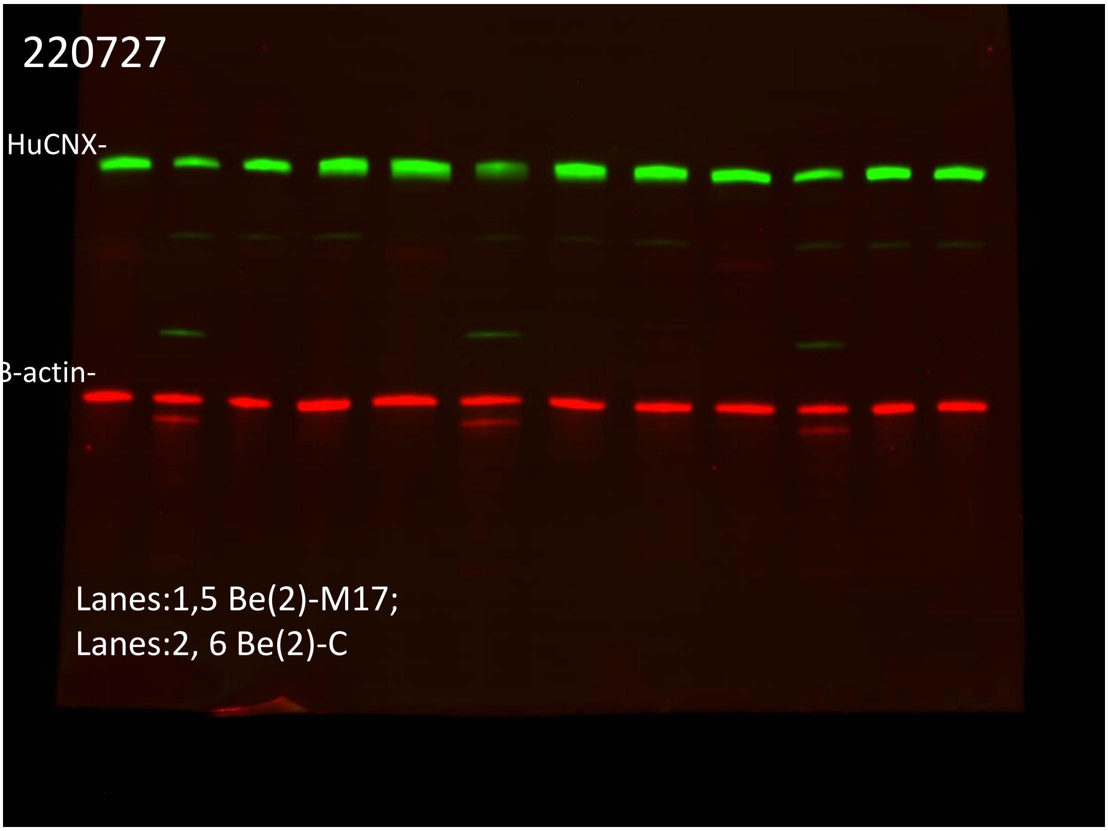
220727
HuCNX-
-actin-
Lanes:1,5 Be(2)-M17;
Lanes:2, 6 Be(2)-C

Human

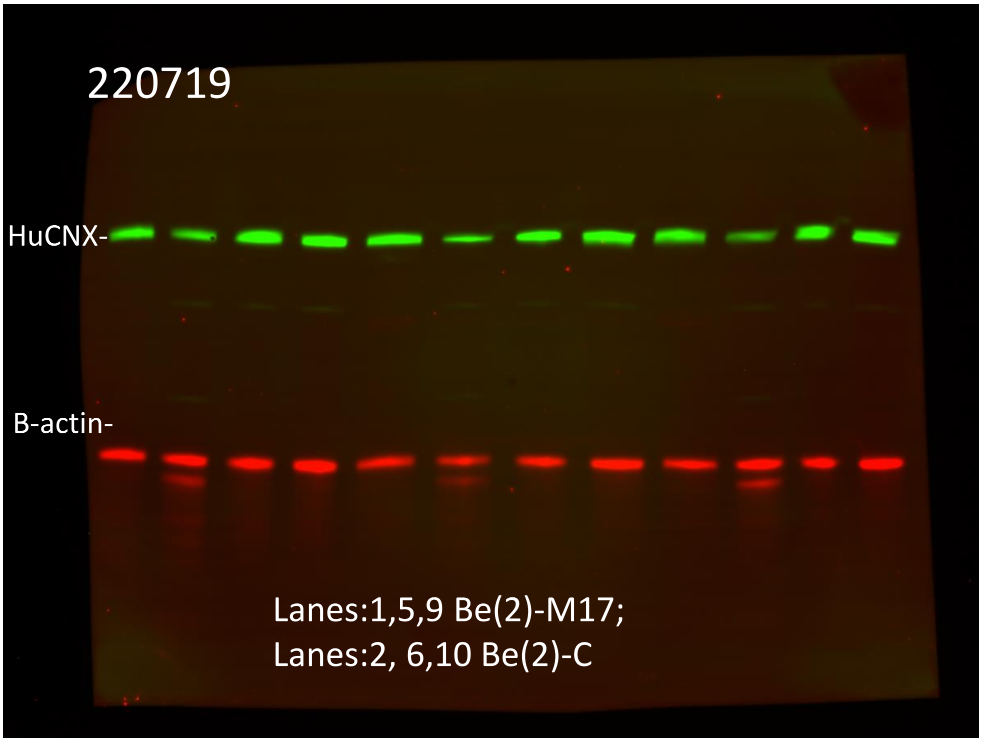
220719
HuCNX-
B-actin-
Lanes:1,5,9 Be(2)-M17;
Lanes:2, 6,10 Be(2)-C

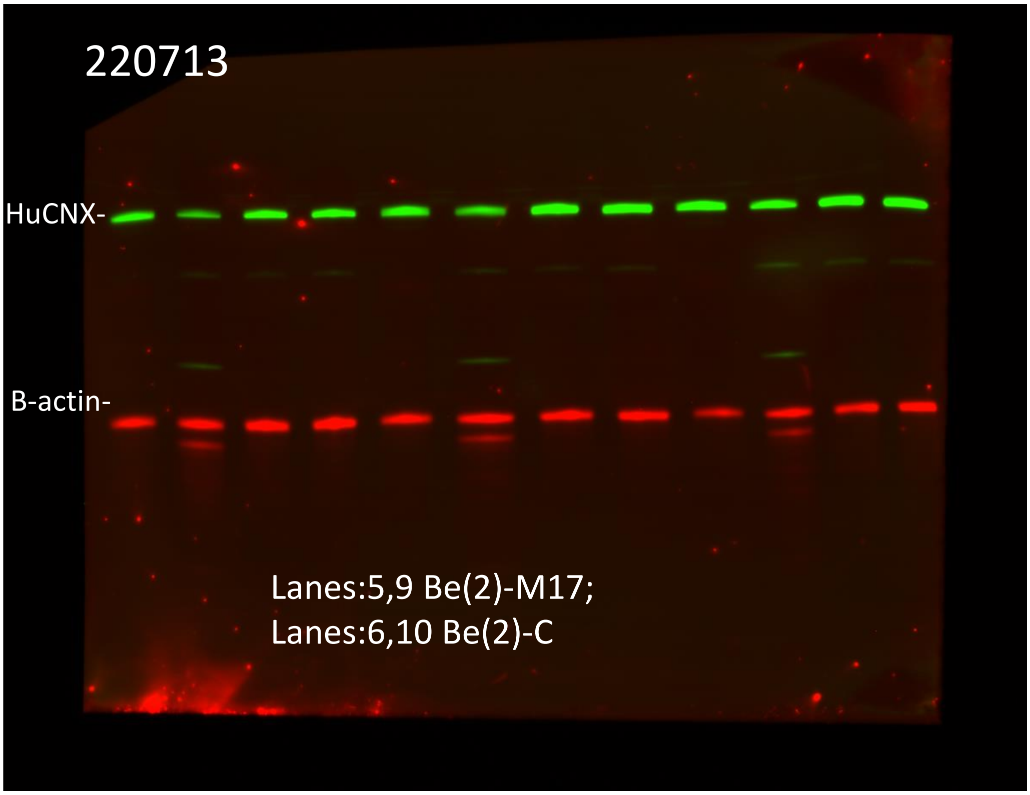
220713
HuCNX-
B-actin-
Lanes:5,9 Be(2)-M17;
Lanes:6,10 Be(2)-C

Human

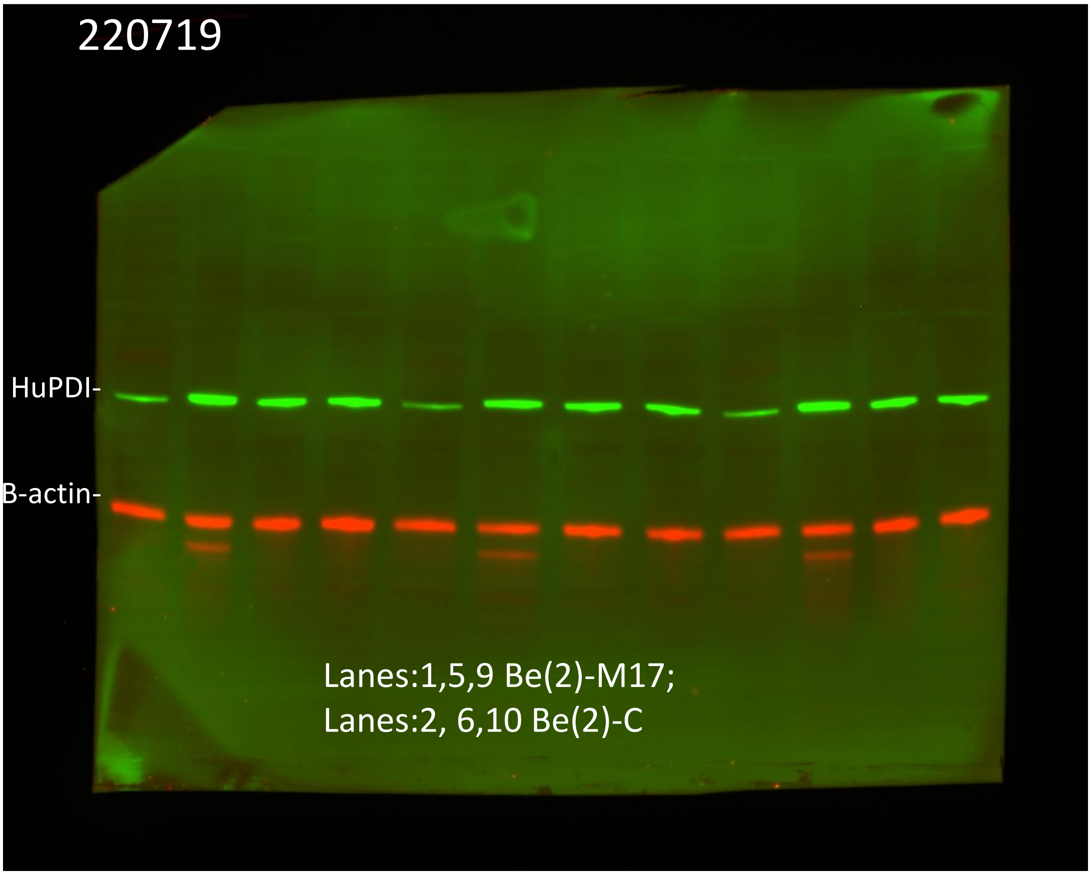
220719
HuPDI-
B-actin-
Lanes:1,5,9 Be(2)-M17;
Lanes:2, 6,10 Be(2)-C

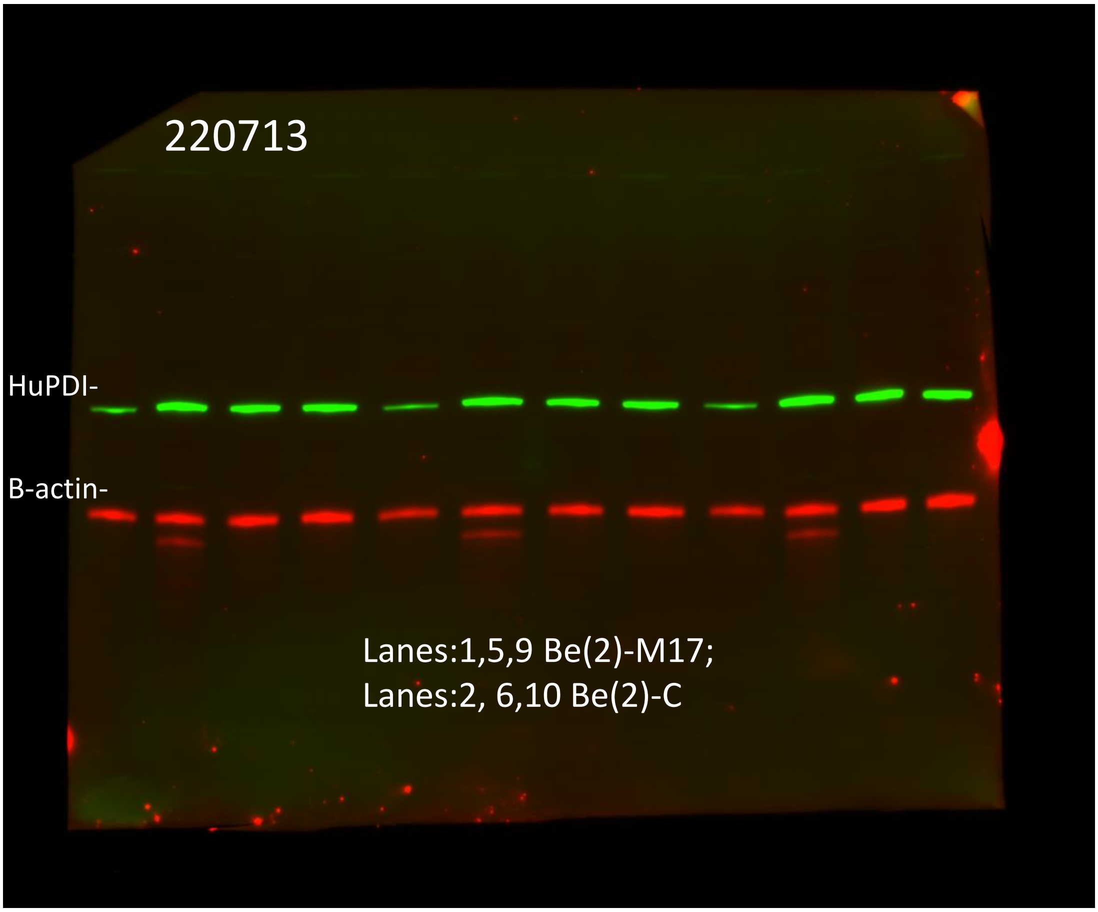
220713
HuPDI-
B-actin-
Lanes:1,5,9 Be(2)-M17;
Lanes:2, 6,10 Be(2)-C

Human

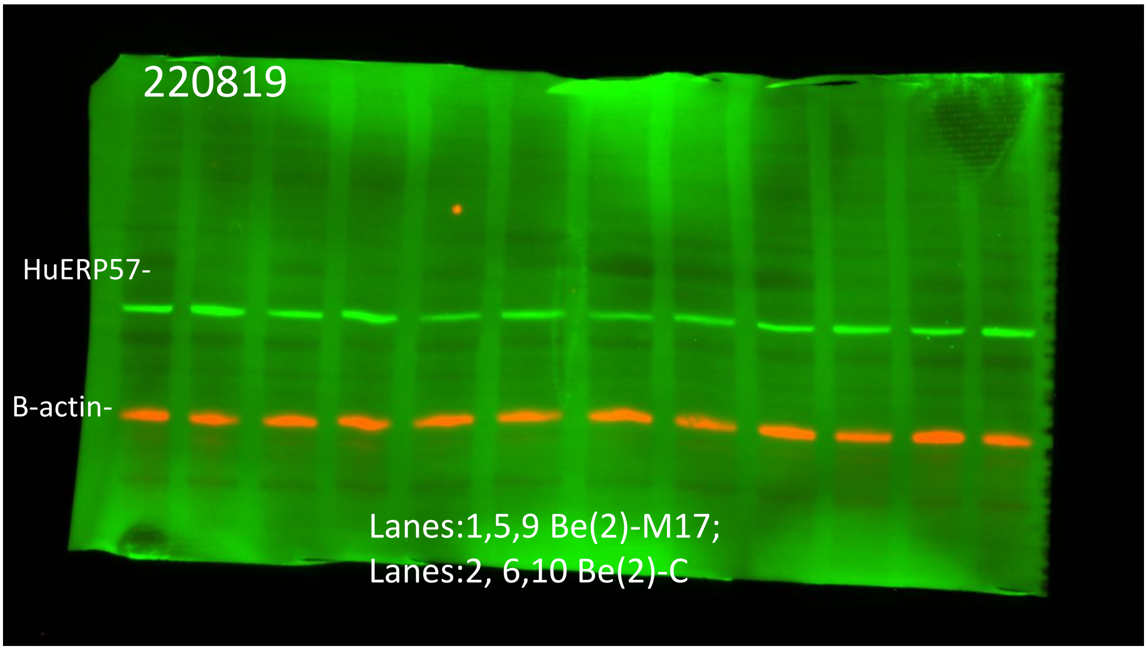
220819
HuERP57-
B-actin-
Lanes:1,5,9 Be(2)-M17;
Lanes:2, 6,10 Be(2)-C

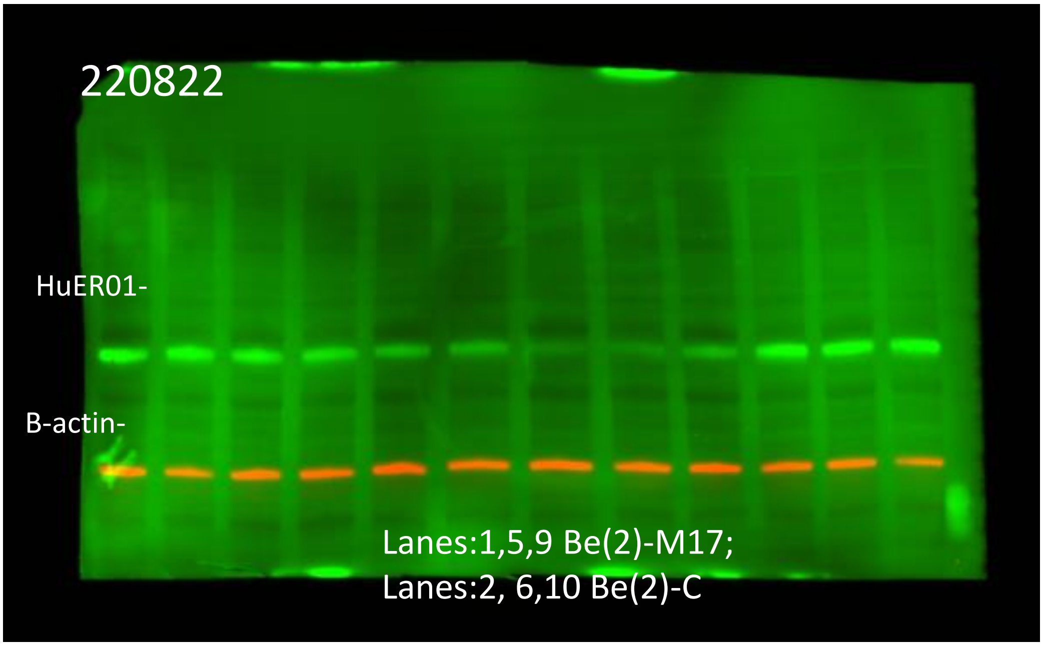
220822
HuER01-
B-actin-
Lanes:1,5,9 Be(2)-M17;
Lanes:2, 6,10 Be(2)-C
